# Endolysosomal Impact of Elevated Ceramide Levels Revealed by Optical and Ultrastructural Nanoprobing

**DOI:** 10.1021/acsnano.5c22213

**Published:** 2026-05-04

**Authors:** Yiqing Feng, Florian Gärber, Essa M. Saied, Harshita Singh, Cecilia Spedalieri, Stephan Werner, Christoph Pratsch, Christoph Arenz, Stephan Seifert, Janina Kneipp

**Affiliations:** † Department of Chemistry, 9373 Humboldt-Universität zu Berlin, Brook-Taylor-Str. 2, 12489 Berlin, Germany; ‡ Einstein Center of Catalysis (EC2/BIG-NSE), 14915 Technische Universität Berlin, Marchstr. 6-8, 10587 Berlin, Germany; § Hamburg School of Food Science, Department of Chemistry, Universität Hamburg, Grindelallee 117, 20146 Hamburg, Germany; ∥ Department X-ray Microscopy, 28340 Helmholtz-Zentrum Berlin für Materialien und Energie GmbH, Albert-Einstein-Str. 15, 12489 Berlin, Germany

**Keywords:** ceramide, surface-enhanced Raman scattering (SERS), gold nanoparticles, random forest, surrogate minimal depth (SMD), acid sphingomyelinase (ASM), N-[(2*S*,3*R*)-1,3-dihydroxyoctadecan-2-yl]2-chloroacetamide (SACLAC)

## Abstract

Ceramide is a central bioactive lipid that acts as both a membrane structural component and a crucial signaling molecule in the cells. In order to maintain cellular homeostasis, ceramide metabolism is tightly regulated by enzymes in the endolysosomal system such as acid ceramidase (AC) and acid sphingomyelinase (ASM). We investigate the biochemical consequence of ceramide accumulation within the endolysosomes of living animal cells by optical nanoprobing, using surface-enhanced Raman scattering (SERS) with gold nanoparticles. The ceramide level in 3T3 fibroblast cells was systematically increased by interfering with two key enzymatic pathways in sphingolipid metabolism as well as by adding exogenous ceramide. The modulation of enzyme activity occurred by the inhibition of AC using the inhibitor N-[(2*S*,3*R*)-1,3-dihydroxyoctadecan-2-yl]­2-chloroacetamide (SACLAC) in different incubation schemes and supplementation of the cells with additional ASM, respectively, both were added through the culture medium. The analysis of SERS data from the endolysosomal compartment reveals changes in the structure and interaction of proteins alongside variations in membrane composition and organization that correspond to ceramide stress. Combined cryo soft-X-ray nanotomography data of the intact cells show that the biomolecular alterations transform the cellular ultrastructure to varying degrees depending on the specific route and extent of ceramide increase. The ultrastructural changes include severe membrane deformation and changed vesicular organization as a consequence of a high ceramide content. The results demonstrate the label-free optical monitoring of metabolic processes at the subcellular level, before their complex biochemical background, and refine the description of molecular and nanostructure changes associated with distorted sphingolipid metabolism.

## Graphical abstract

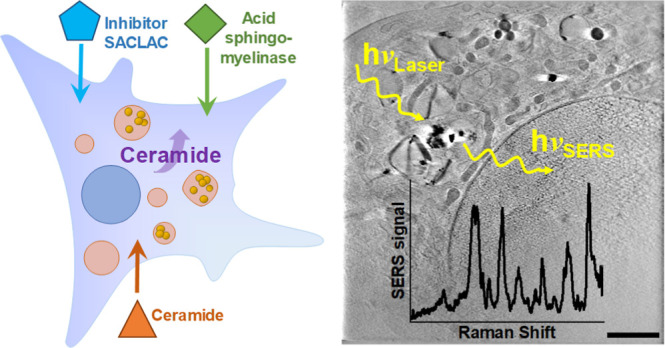

Ceramide is a crucial lipid molecule, as it has functions as both an important structural building block and as a second messenger in animal cells. As a fundamental building block of the cell membranes, ceramide can drastically reorganize membrane structure, change permeability, and facilitate signal transmission through lipid raft formation.^1–3^ It is also a potent bioactive signaling lipid involved in cellular stress responses, senescence, and apoptosis.^4^ At the center of sphingolipid metabolism, ceramide acts as a precursor to synthesize complex sphingolipids, and it is also a product of their catabolism.^4,5^ The balance of intracellular ceramide, as a core component of sphingolipid homeostasis, must be tightly regulated, so that essential cellular processes are maintained.^5^ The intracellular regulation of ceramide involves the coordination of multiple enzymes, including two important lysosomal molecules, acid ceramidase (AC) and acid sphingomyelinase (ASM).^5,6^ At the optimal pH range between pH4.5 and pH5, AC catabolizes ceramide into sphingosine and free fatty acids, whereas ASM generates ceramide via the hydrolysis of sphingomyelin,^5,6^ thereby dynamically controlling the balance of ceramide in the lysosomes. When these metabolic pathways are perturbed, for example, through a genetic defect,^7,8^ environmental stress,^9^ or enzymatic inhibition,^10,11^ the fluctuation of ceramide content in the lysosomes can lead to a series of significant downstream influences. Excess ceramide has been shown to impact organelle membrane properties,^12,13^ as well as to interfere with key signaling pathways.^14^ Especially in the lysosomes, where essential cellular catabolism and metabolic processes are regulated,^15^ ceramide accumulation impairs the membrane integrity^16^ and induces dysfunction of the whole organelle.^17^ These changes can trigger additional pathologies in patients, including lysosomal storage disorders such as Farber disease^18^ or Niemann-Pick disease.^19^

In this study, we probe the molecular changes under ceramide stress in the endolysosomal system of living 3T3 fibroblast cells in a label-free way. Vibrational nanospectroscopy identifies the local composition, biomolecular structure, and molecule–molecule interactions from all types of molecules, and in volumes that correspond to subcellular environments, and exhibits strong potential to capture mild molecular changes in living cells.^20–23^ By using gold nanoparticles as probes, surface-enhanced Raman scattering (SERS) has shown a high sensitivity for a close observation of biomolecular interaction and dynamics in the endolysosomal compartment under distinct physiological conditions.^24–28^ While Raman microspectra are typically collected from diffraction-limited focal volumes on the order of tens of femtoliters, the high enhancement and localization of the SERS signals enable the observation of individual SERS-probe containing endolysosomes and the nanoscopic volumes in them. The SERS signals originate in the immediate, few-nanometer vicinity of the nanoprobes. Given the complex biological matrices of cellular models, integration of SERS with efficient machine learning algorithms is crucial to interpret the fingerprint-like vibrational information.^29^ Specifically, the Random forest (RF)-based approach of surrogate minimal depth (SMD) has become a powerful tool to investigate the colocalization of molecular groups^11,30,31^ and thereby can reveal subtle biochemical alterations.^32^ SMD integrates variable relationships into the selection of important spectral features and enables the analysis of feature relationships based on their mutual impact on the RF model. This approach has already been successfully applied to many different types of data,^33–35^ including the analysis of relationships across different kinds of analytical data.^36–39^

Here, we discuss the molecular and ultrastructural effects of elevated intracellular ceramide levels based on endolysosomal probing. Three different, independent pathways were used to increase the amount of ceramide in the cells: (i) the irreversible inhibition of AC by *N*-[(2*S*,3*R*)-1,3-dihydroxyoctadecan-2-yl]­2-chloroacetamide (SACLAC), an alpha-chloroamide ceramide analogue,^40^ (ii) supplementation of the cells with additional, external ASM enzyme, and (iii) the addition of exogenous ceramide to the cultured cells. To infer the structure and composition in the endolysosomal environment, we analyze the occurrence of vibrational SERS signals that are selectively originating from this organelle. We demonstrate that SMD can be applied to analyze and compare the colocalization of specific biomolecular components and structures in cells undergoing different control of ceramide content. As will be discussed, the observation of colocalizing spectral features is used to identify distinct patterns of molecular interactions and structure alterations that are characteristic of the type of manipulation of ceramide content, the time point of endosome formation, and ceramide dose. The molecular information from the live cells will be discussed in the context of changes in the cellular ultrastructure, as observed by soft X-ray nanotomography (SXT) of the intact cells. As will be demonstrated, the data facilitate our understanding of subtle subcellular responses to ceramide stress originating from diverse pathways.

## Results and Discussion

### Mature Endolysosomes Adapt to Sudden Ceramide Build-Up when AC Is Inhibited

In our experiments, we probe the endolysosomal composition by SERS in the situations of (i) inhibited breakdown, (ii) increased synthesis, and (iii) additional, internalized ceramide, respectively. In total, 40,800 spectra were collected from cells under ceramide stress and their controls. Scheme 1 gives an overview of the respective incubation conditions of the cultured cells. The 3T3 fibroblast cells were incubated with the inhibitor SACLAC in two different ways. In the first experiment (Scheme 1A), the cells were first incubated with the gold nanoprobes that are taken up into the endolysosomal system and that cause strong SERS signals from the endolysosomal environment. In the next incubation step, the inhibitor SACLAC was added in two concentrations. Thereby, the influence of the enzyme inhibitor, expected to lead to a buildup of ceramide in the pre-existing, matured an endolysosomal environment, is studied (Scheme 1A). At the time of probing, all of the SERS probes are contained in lysosomal structures. In a second set of experiments, the incubation sequence was reversed (Scheme 1B): an incubation with SACLAC before the gold nanostructures enables SERS probing of endolysosomes that have formed under the influence of higher ceramide levels.

**Scheme 1:**
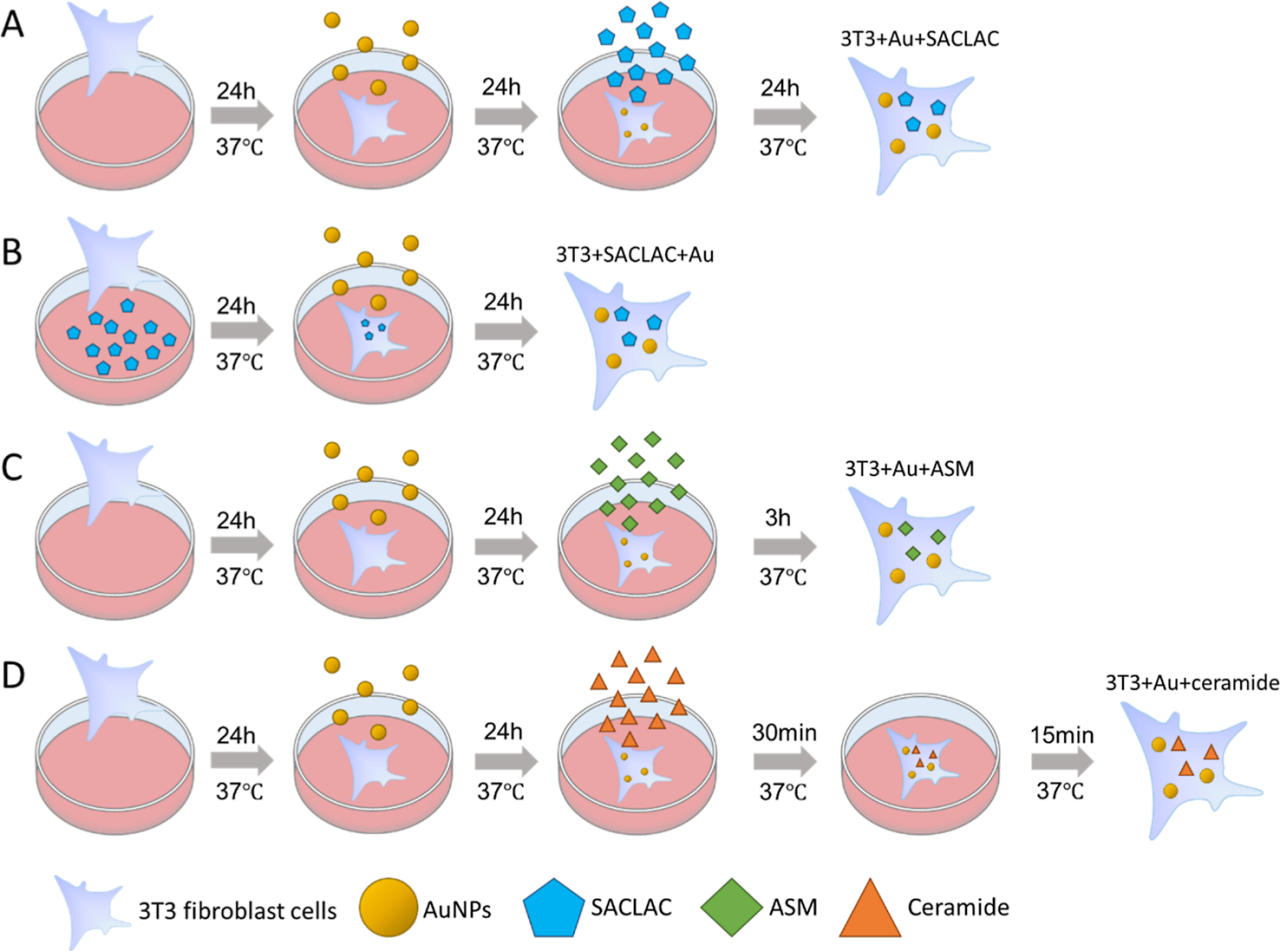
Schematic for the Incubation of 3T3 Cells with Gold Nanoparticles, SACLAC, ASM, and Ceramide for SERS Experiments. (A) Cells Were Incubated with SACLAC for 24 h after the Incubation with Gold Nanoparticles for 24 h; (B) Cells Were Seeded in the Culture Medium with SACLAC and Incubated for 24 h Prior to the Incubation of Gold Nanoparticles for 24 h; (C) Cells Were Incubated with 200 nM ASM for 3h after the Incubation with Gold Nanoparticles for 24 h; and (D) Cells Were Incubated with Ceramide for 30 min after the Incubation with Gold Nanoparticles for 24 h

The toxicity of SACLAC was assessed by determining cell viability by an XTT cell proliferation assay, applying the inhibitor in seven concentrations (Figure S2). The inhibitor concentration was chosen in such a way that the viability is not drastically decreased, but effects are visible. The SACLAC treatments of the cells at 1 and 10 μM in the culture medium reduced the viability to 91% and 87%, respectively (Figure S2).

The SERS spectra of the SACLAC-treated cells together with a statistical analysis of the occurrence of particular signals in the data sets that typically comprise several hundreds of spectra are displayed in Figure 1. A summary of tentative assignments of the vibrational bands is found in Table S1. The data reveal the influence of increased ceramide in mature endolysosomes, when incubation occurs according to Scheme 1A (Figure 1A to Figure 1D) (for data from controls, see Figure S1A–D). All treated and control groups share a number of spectral features, mostly assigned to proteins (cf. Table S1), specifically signals of the amide groups at 1704, 1276, and 1230 cm^–1^,^11,28,41–43^ of different individual amino acids at 1347, 870, 837, and 755 cm^–1^,^11,28,41–45^ of a mode assigned to protein backbone C–C/C–N stretching at 1132 cm^–1^,^42,44^ and signals of disulfide groups at 658, 553, and 503 cm^–1^.^11,28,42,44,46,47^ A deformation mode of lipid CH_2_/CH_3_ groups at 1318 cm^–1^ ^30,42^ is a common feature in all samples as well.

**Figure 1:**
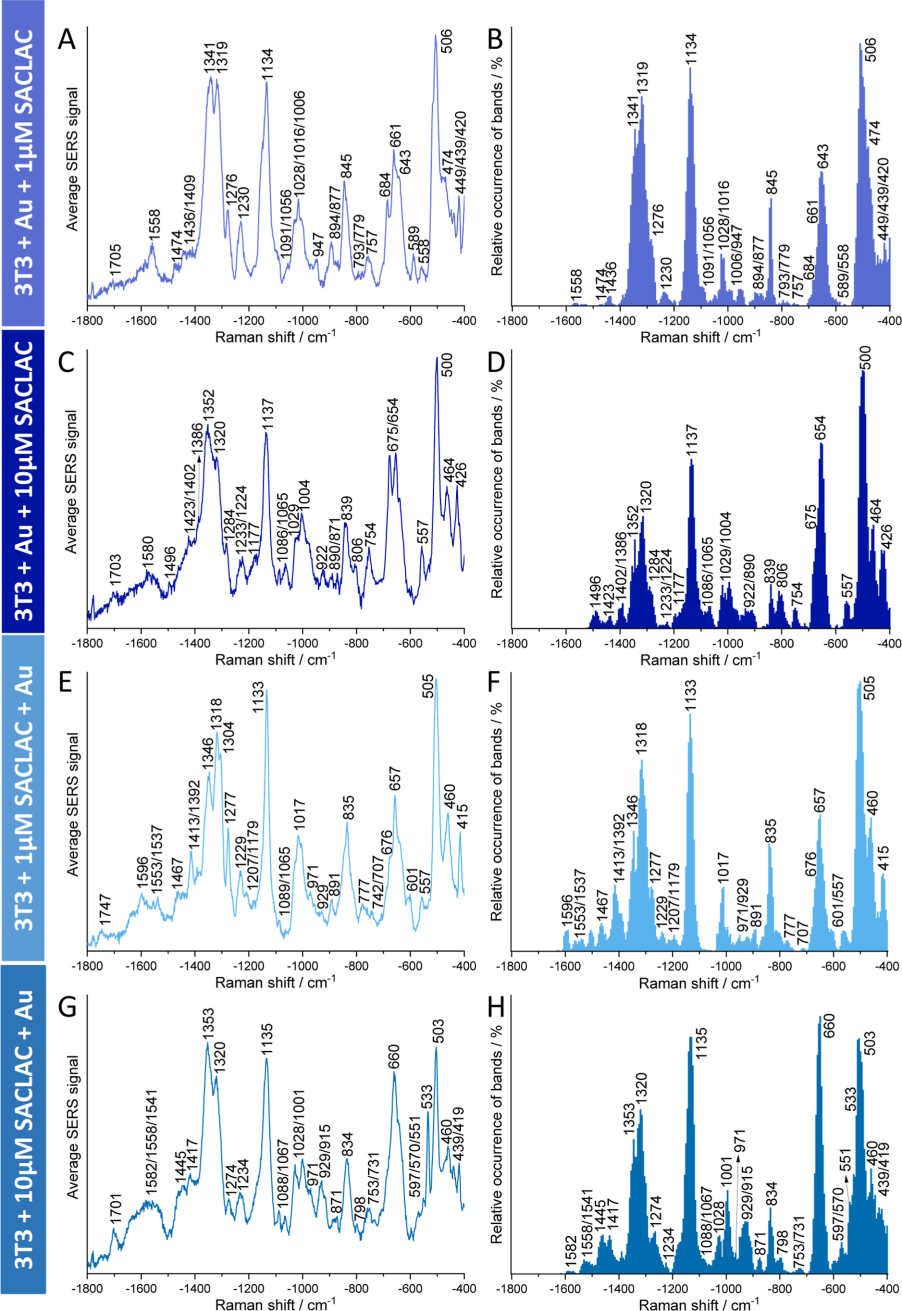
Average SERS spectra (A,C,E,G) and relative band occurrence (B,D,F,H) of the respective SERS data sets of 3T3 cells incubated with (A,B) 1 μM SACLAC and (C,D) 10 μM SACLAC for 24 h after incubation with gold nanoparticles for 24 h (cf. Scheme 1A) of 3T3 cells incubated with gold nanoparticles for 24 h after seeding in the culture medium with (E,F) 1 μM SACLAC and (G,H) 10 μM SACLAC for 24 h (cf. Scheme 1B). The data sets contain 366 (A,B), 770 (C,D), 550 (E,F), and 1007 (G,H) SERS spectra, respectively. Excitation wavelength: 785 nm. Excitation intensity: 2.7 × 10^5^ W cm^–2^. Acquisition time: 1 s.

When SACLAC was added to the cells at the lower concentration of 1 μM (Figure 1A,B), several spectral features indicate changes in the conformation of proteins in the endolysosomal environment compared to the untreated control cells (cf. Figure S1A,B). Specifically, the amide II band at 1574 cm^–1^ ^28,42^ and a stretching mode of disulfide bridges at 465 cm^–1^ ^28,44^ are not observed, along with increased occurrence of signals from an amide III mode at 1276 cm^–1^.^28,42,43^ The altered protein structure and interaction is also evidenced by the increased presence of signals of aromatic protein side chains that are exposed differently, particularly of phenylalanine at 1028 and 1006 cm^–1^ ^11,42,43,47–49^ and a shift of the tyrosine ring breathing mode from 838 cm^–1^ to 845 cm^–1^ ^11,41,43,45,49^ in the SACLAC-treated cells (Figure 1A,B). Moreover, new vibrational bands at 1436, 1056, and 779 cm^–1^, assigned to lipid CH_2_ deformation modes,^41,43^ acyl chain C–C stretching modes,^43,47,50,51^ and phosphatidylinositol,^41,43^ respectively, are found in the endolysosome spectra of the treated cells. A pronounced signal at 1389 cm^–1^ assigned to a deformation of CH_3_ groups^43^ is absent compared to the control samples (Figure S1A). Although the very specific SERS signals of pure ceramide^52^ are not found here, the changes of the lipid signals suggest a reorganization of membrane composition and structures under the influence of the additional ceramide that are well in-line with structural features observed in previous studies of liposomal membrane models, including ceramide containing liposomes.^52,53^ It should be noted that the viability of the cells exposed to 1 μM SACLAC is only slightly decreased, as determined from the XTT test (Figure S2). The observed molecular changes suggest that the processes induced by SACLAC at this minimally toxic concentration are part of an adaptation to the accumulation of ceramide.

At a higher SACLAC concentration of 10 μM, viability drops to ∼ 87% (Figure S2). There, the endolysosomal environment undergoes more complex changes (Figure 1C,D). Specifically, additional signals indicate significant changes in a protein secondary structure, represented, e.g., by the amide II and III bands at 1497 and 1224 cm^–1^,^11,28,43,44^ respectively, and the presence of an amino acid carboxylate stretching vibration at 1402 cm^–1^ ^11,45^ that is absent from the spectra of the controls (Figure S1C,D).

The spectral changes in the signals of lipids are much more pronounced after exposure of the cells to this higher concentration of SACLAC. Specifically, a bending mode of lipid CH_3_ groups at 1386 cm^–1^,^43^ a phosphate group vibration at 1180 cm^–1^,^11,30^ and the frequent occurrence of a cholesterol mode at 426 cm^–1^,^43^ together with the absent or less frequent signals of different lipid CH_2_ deformation modes reveal severe changes of lipid state-of-order and membrane fluidity.^51^ The lower cell viability (Figure S2) is in agreement with the higher toxicity of SACLAC at increased concentration that was reported in human acute myeloid leukemia cell lines.^10^ Our data indicate that the toxic action must be linked to the drastic structural changes of the lipid membranes observed here. The pronounced changes indicate a dose-dependence of the ability of the fibroblast cells to adjust the molecular environment of mature endolysosomes in response to an accumulation of ceramide when the usual breakdown of the molecule by AC is impaired.

### Probing of Endolysosome Development and Aging in a Ceramide-Rich Environment

In the spectral analysis following incubation according to Scheme 1B, spectra are collected from endolysosomes that formed when SACLAC had already inhibited AC and altered the acting of the lipid metabolism of the cells (Figure 1E–H). In these experiments, the SERS nanoprobes are enclosed in endolysosomes that form and develop in a ceramide-rich environment. The SERS spectra of all cells exhibit several common vibrational bands associated with protein and lipid interactions with the nanoprobes (compare Figures 1 and S1), irrespective of an inhibition of AC. They include important signals that are associated with the lipid acyl chains and vibrations of proline, valine, and tryptophan (Figure 1, Table S1). We assign the occurrence of these spectral features to the different endolysosomal environment, including the processing of the protein corona that reaches different stages in the long continuous incubation period of 24 h.^44^

A number of spectral differences between the control and SACLAC-treated cells were found to be independent of SACLAC concentration (Figure 1E–H). In cells treated with either 1 or 10 μM SACLAC before the incubation with the nanoprobes, the amide II signals are modified, with a band at 1527 cm^–1^ ^28^ in the control samples (Figure S1E,F) being replaced by new amide II components at 1555 and 1539 cm^–1^ ^11,28,30,42,43,51,54^ (Figure 1E–H, Table S1). Together with the loss of signals in the amide III band at 1221 cm^–1^, associated with β-sheet structures,^28,43^ and other signals suggesting less and differently stabilized disulfide bridges,^55^ they indicate alterations in protein conformation in the endolysosomes upon SACLAC treatment and the induced endolysosomal stress.

The changes found with respect to the amino acid side chains as a consequence of protein structural alterations vary in the experiments with the two concentrations of the inhibitor. At a SACLAC concentration of 1 μM, bands of the aromatic amino acids, tyrosine, tryptophan, and phenylalanine occur much less frequently than in the control spectra, and phenylalanine is represented by a C=C deformation band at 1596 cm^–1^ ^43,49,56^ in addition to its ring breathing vibrations in the range from 1000 to 1030 cm^–1^ (compare Figures 1E with S1E, Table S1). Under 10 μM SACLAC treatment, exclusive appearances of tryptophan at 753 and 570 cm^–1^ ^28,41,43,44^ indicate an altered microenvironment of tryptophan residues (compare Figures 1G and S1E).

As a result of the SACLAC-induced increase in ceramide, changes of lipid signals were observed. The frequently occurring band assigned to a CH_2_ deformation of lipid chains at 1429 cm^–1^ ^28,42,57^ that is present in the control spectra (Figure S1E,F) disappeared in the treated cells, in agreement with the results found when SACLAC acted exclusively on mature endolysosomes discussed above (Scheme 1A). In the cells treated with 1 μM SACLAC, signals of the acyl chains occur less frequently (Figure 1F) than in the control cells (Figure S1F). When cells were exposed to 10 μM SACLAC, even more differences were found. They include the shift of another CH_2_ deformation band from 1460 cm^–1^ to 1445 cm^–1^ ^11,42–44^ (Figure 1G), an additional lipid acyl chain C–C stretching band at 1065 cm^–1^ ^43,47,50,51^ (Figure 1E,G) and also the occurrence of a signal from cholesterol at 439 cm^–1^.^11^ All these changes indicate that the ceramide enrichment in the endolysosomes affects lipid composition, membrane packing, and fluidity in this compartment.

### Effects of AC Inhibition Vary for Different Inhibitor Incubation and Probing Time Points

Both treatments with the inhibitor SACLAC, prior to or after nanoprobe exposure (Scheme 1A,B, respectively), lead to ceramide accumulation in the endolysosomes through AC inhibition. The data of Figure 1 show that the effects on endolysosomal molecular composition and structure at two inhibitor doses, both with only moderately decreasing viability (Figure S2), differ depending on the relative timing of nanoparticle internalization and inhibitor exposure. The effects of the different conditions at which the inhibitor was added to the cell cultures with respect to the control group are visible in the score plots of a principal component (PC) analysis (PCA) (Figure S3A,C,E,G). The plots indicate the overall dissimilarity of the data in each condition. The molecular variation with respect to the onset of ceramide increase (Scheme 1A vs Scheme 1B) is represented qualitatively in the PC loadings of a PCA of each data set (Figure S3B,D,F,H). The scores of the first two principal components (PC1 and PC2) exhibited an extensive overlap of the data from all SACLAC-treated and control cells, in agreement with the abundance in common spectral features discussed above and their general cell-to-cell variation in both groups (Figure S3A,C,E,G). As revealed by a comparison of the loading spectra (Figure S3B,D with S3F and S3H, respectively), in full agreement with the discussed band occurrence in Figure 1 (cf. Figure 1B,D with Figure 1F,H, respectively), the variation in lipid organization exhibited marked differences upon the two probing regimes. As an example, an absence of an influence on CH_2_ deformation vibrations under SACLAC treatments following nanoparticles (Figure S3B,D) likely reflects an adaptation of membrane structures in the existing more mature endolysosomes (cf. Scheme 1A). In contrast, an earlier exposure of SACLAC, prior to gold nanoprobe uptake (cf. Scheme 1B, Figure 1F,H, loadings in Figure S3F,H), shows a difference with respect to lipid chain organization by contributions around 1440 cm^–1^ and supports that the alterations due to ceramide build-up have established before probing. Contributions from cholesterol signals that are different from the controls were observed for both incubation schemes, when the higher SACLAC concentration of 10 μM was administered (Figure 1D,H). However, the bands from cholesterol presented at 439 cm^–1^ ^11^ (Figure 1D) and 426 cm^–1^ ^43^ (Figure 1H), respectively, indicating a different effect on membrane stability, depending on the situation under which the endolysosome has formed. This is in agreement with the stabilizing function of cholesterol in sphingolipid membranes.^58^ Moreover, different effects of SACLAC-induced ceramide enrichment on the conformation of endolysosomal proteins were observed. Adding SACLAC before gold nanoparticles (Scheme 1B) displayed more secondary structure modifications, visible, e.g., in altered amide II bands. Differently, spectral differences when SACLAC was added after the nanoprobes (Scheme 1A) are mainly associated with backbone vibrations and signals from certain amino acid side chains. It suggests that the alterations of protein structures are more localized, when the matured, more complex endolysosomes (as in the experiments according to Scheme 1A) must adapt to increased ceramide content.

The vibrational distinctions induced by endolysosomal ceramide build-up were found to be dependent on SACLAC dose for both incubation regimes (Scheme 1A,B). In the cells treated with 1 μM SACLAC, C–S bands occur (Figure 1F,B) less frequently, indicating more stable disulfide bridges in proteins.^44,46^ In contrast, changes in amino acids and lipids are more evident in the cells with 10 μM SACLAC (Figure 1D,H), which implies more complex influences on the endolysosomal environment at this inhibitor concentration. These dose-dependent effects suggest that biophysical changes in endolysosomes are more pronounced with increasing ceramide level.

### Endolysosomal Adaptation to Ceramide Enrichment by Addition of Acid Sphingomyelinase

The ceramide accumulation through the enhanced production of ceramide by the enzyme ASM was investigated by administering additional ASM through the cell culture medium. To ensure optimum activity of ASM throughout the experiment, 3T3 cells were incubated with the enzyme for 3 h after the incubation with the optical nanoprobes for 24 h (Scheme 1C). Their SERS data are shown in Figure 2A,B (control data in Figure 2C,D). High similarity was observed in the SERS spectra of both ASM-treated cells and control samples (Figure 2A,C), in agreement with the substantial overlap of PCA clusters (score plot in Figure S4A). It is interesting to note that the PC1 loading (upper trace in Figure S4B) highly resembles those from the SACLAC-exposed cells (upper traces in Figure S3B,D,F,H), consistent with common, predominant contributions from proteins and lipids to the spectra. Pronounced phenylalanine signals at 1028 cm^–1^ ^43,47–49^ and 1000 cm^–1^ ^11,42,43,49^ were found in both ASM-treated and control cells (Figure 2A,C).

**Figure 2:**
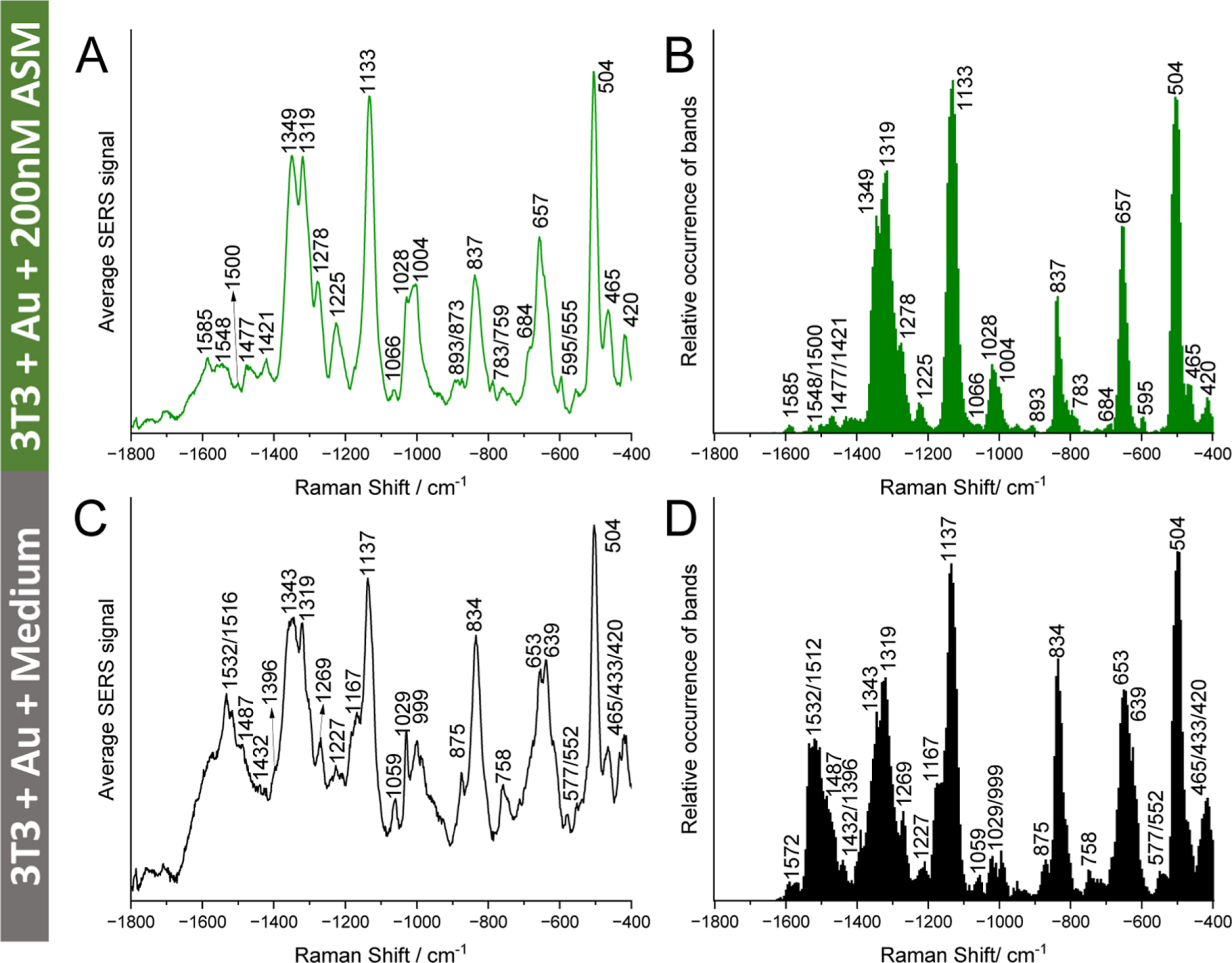
Average SERS spectra (A,C) and relative band occurrence (B,D) of the respective SERS data sets of 3T3 cells incubated with (A,B) 200 nM ASM for 3 h after incubation with gold nanoparticles for 24 h (cf. Scheme 1C) and (C,D) control samples only incubated with gold nanoparticles for 24 h. The data sets contain 1050 (A,B) and 1251 (C,D) SERS spectra, respectively. Excitation wavelength: 785 nm. Excitation intensity: 2.7 × 10^5^ W cm^–2^. Acquisition time: 1 s.

Compared to the spectra from the control cells (Figure 2C,D), amide II signals at 1500, 1548, and 1585 cm^–1^,^28,30,42,43^ which appeared at higher vibrational frequencies, presented less often in the cells exposed to additional ASM (Figure 2A,B). These marked changes of protein secondary structure, induced by increased ceramide, are also evidenced by signals from amino acids that indicate different burial of side chain residues in control and ASM-treated cells (compare Figure 2A with C, Table S1).^11,28,41–45,49,59^

The data indicate important changes of lipid composition and structure in the cells exposed to additional ASM, specifically the absence of a cholesterol signal at 433 cm^–1^ ^11^ and new signals of phosphatidylinositol vibrations at 783 cm^–1^ and 595 cm^–1^ ^41,43^ (Figure 2A,B). These changes of the composition of the membranes,^41,43,60^ are accompanied by more dramatic membrane reconstructions, indicated, e.g., by fewer signals of the lipid intrachain C–C stretching vibration at 1059 cm^–1^ ^43,47,50,51^ (Figure 2B) and a shift of the lipid CH_2_ deformation vibration from 1432 cm^–1^ ^41,43^ (Figure 2C) to 1477 cm^–1^ ^28^ (Figure 2A). Both vibrations point to alterations of membrane packing and state-of-order under the ASM treatment.

Although the major spectral variation appears similar when ceramide accumulation is induced by different pathways, i.e., by inhibition of AC or addition of ASM (cf. loadings in Figures S3 and S4, respectively), several of the changes are very distinct and characteristic of the addition of ASM. Especially, the observed drastic alteration of lipid organization is probably due to the rapid increase in ceramide content in the membranes.^58,61^ A 2-fold increase in total ceramide within 30 min was reported in human mammary carcinoma cells upon activation of the ASM/ceramide pathway by exposure to cisplatin.^62^ In contrast, inhibition of AC by SACLAC was shown to induce ceramide accumulation in a more progressing fashion.^18^ The results observed here indicate a reduced stability of the membranes, evidenced, e.g., by the disappearing signal of cholesterol, and are in agreement with the drastic changes to lipid composition found in other cell types.^58,61,62^

Although some of the alterations to protein structure show similarities to effects of AC inhibition by SACLAC, the specific absence of tyrosine and tryptophan signals when ceramide content increases as a consequence of additional ASM implies that distinct microenvironments of aromatic residues are influenced when this metabolic pathway is affected. While both SACLAC and ASM are known to enrich ceramide, they have different effects on protein and lipid structure and composition in the endolysosomal environment, especially remarkably different impacts of ceramide excess on the membranes.

### RF Analyses Reveal Changes in Molecular Interactions upon Inhibition of AC

The supervised approach of RF was applied to assess if the spectra from SACLAC-treated cells can be differentiated from those of control samples. The RF models achieved classification accuracies higher than 80% for both training and test data sets (Table S2), indicating that distinguishable molecular patterns exist between treated cells and control samples, despite several similarities. SMD was applied to select the spectral features that contribute to differentiation (Figure S5), and the SMD relation parameter mean adjusted agreement (MAA) was calculated between the selected, important bands and all other spectral variables to analyze the co-occurrence of spectral features, as indicators of molecular colocalization and interaction (Figure 3). By the application of RF-SMD analysis, several differences between the treated and control cells become obvious that are not observed in the band occurrence analysis shown in Figures 1 and 2.

**Figure 3:**
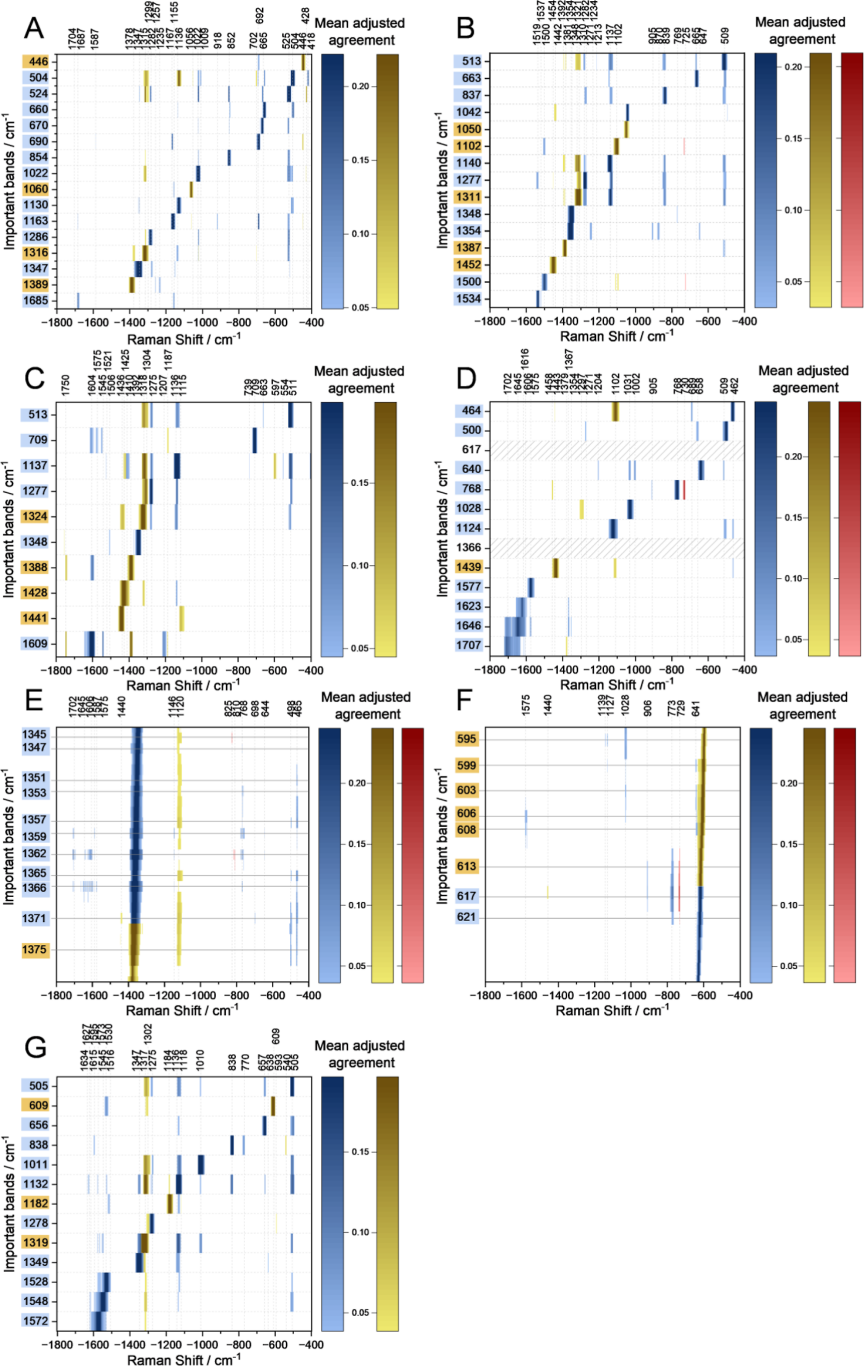
Spectral variables across the spectral range that co-occur with the important bands (left) selected by SMD analysis (cf. Figure S5) of SERS spectra from 3T3 cells treated with (A) 1 μM SACLAC and (B) 10 μM SACLAC for 24 h after incubation with gold nanoparticles for 24 h (cf. Scheme 1A), with (C) 1 μM SACLAC and (D–F) 10 μM SACLAC for 24 h prior to 24 h incubation of gold nanoparticles (cf. Scheme 1B), and with (G) 200 nM ASM for 3 h after incubation with gold nanoparticles for 24 h (cf. Scheme 1C). The striped rows in (D) correspond to two broad SMD features in Figure S5D, and their details are illustrated (E) between 1344 and 1380 cm^–1^ and (F) between 592 and 630 cm^–1^. The data sets contain 366 (A), 770 (B), 550 (C), 1007 (D–F), and 1050 (G) SERS spectra, respectively, after elimination of spectra with no signal after the experiment. Bands assigned to proteins, lipids, and DNA are marked in blue, yellow, and red, respectively. SMD values are shown in Figure S5.

As the analysis of the 1 μM SACLAC treatment-after-nanoprobe incubation shows (Figure 3A), differences due to cholesterol are already found at low SACLAC concentration, and its bands at 702, 446, and 428 cm^–1^ (Figure 3A, yellow blocks in the map)^11,41,43^ were highly associated with protein signals at 1163, 1022, 690, 524, and 504 cm^–1^ (Figure 3A, blue important bands on the left).^11,28,41–44,46–49,59^ The complex network of related signals, particularly from lipids at 1315 cm^–1^ ^30,42^ , cholesterol at 428 cm^–1^ ^49^, amino acids at 1022 cm^–1^ ^43,47–49^ and 852 cm^–1^,^49^ and the protein C–C/C–N bond vibration at 1129 cm^–1^ (Figure 3A, blue and yellow blocks in the map) co-occurred with disulfide vibrations at 524 cm^–1^ ^43,59^ and 504 cm^–1^ (Figure 3A, important bands in blue).^11,28,42,44,46^ The known sensitivity of several of the discussed protein signals to changes in protein secondary structure indicates that the protein conformation near cholesterol-rich domains is changed when the ceramide level increases. It is likely that membrane reorganization, mediated by ceramide enrichment, alters protein stability.

When the concentration of SACLAC in the experiments was increased to 10 μM (Figure 3B), further important indicators of the protein structure and stability become visible. The disulfide stretching vibration at 509 cm^–1^ ^11,28,42,44,46^ exhibits a different network of co-occurring signals,^30,42^ including components of the amide III region at 1271–1282 cm^–1^ ^28,42,43^ and a shifted protein backbone vibration at 1137 cm^–1^ (Figure 3B, blue blocks in the map).^42,44^ This implies that distinct protein unfolding occurred as a consequence of AC inhibition. Notably, the tryptophan vibration at 1354 cm^–1^ (Figure 3B, blue important band on the left)^11,28,42,44^ shows high relations with the amide III component at 1234 cm^–1^ ^11,42,43^ and several amino acid bands, e.g., at 905 cm^–1^,^28^ 870 cm^–1^,^28^ and 647 cm^–1^ ^41,49,59^ (Figure 3B, blue blocks in the map), while a tryptophan signal at 1348 cm^–1^ (Figure 3B, blue important band on the left) co-occurs with the ring breathing mode of the molecule at 769 cm^–1^ ^43,63^ (Figure 3B, blue block in the map), indicating distinct populations of tryptophan residues with different physicochemical environments that underpin the changes in protein structure.

In endolysosomes that are formed during the ongoing inhibition of the AC enzyme (Scheme 1B), co-occurrence of a signal of phosphatidylinositol at 597 cm^–1^ ^43^ (Figure 3C, yellow block in the map) with the protein backbone vibration at 1137 cm^–1^ ^42,44^ (Figure 3C, blue important band on the left) is found for low inhibitor concentration. This is consistent with the known ability of phosphoinositide to recruit effector proteins at endolysosomal membranes.^60^ A strong association of the phenylalanine vibration at 1609 cm^–1^ ^43,49,56^ and a deformation at 1388 cm^–1^ assigned to lipid tails^11,43^ (Figure 3C, blue and yellow important bands on the left) indicates a different exposure of phenylalanine protein side chains near lipid membranes in the cells with high ceramide content compared to the control samples. These changes are further underpinned by lipid signals that indicate an increased disorder of lipids, specifically a band at 1115 cm^–1^ (Figure 3C, yellow block in the map), assigned to lipid intrachain gauche conformers,^11,43,50^ and its close relation to the methylene deformation at 1441 cm^–1^ ^11,42–44,50^ (Figure 3C, yellow important band on the left). The other CH _2_ deformation mode at 1428 cm^–1^ ^28,42,57^ (Figure 3C, yellow block in the map), which is found often in the control samples (Figure S1F), is highly related to the lipid CH_2_/CH_3_ deformation vibration at 1311 cm^–1^ ^30,42^ and a protein backbone mode at 1140 cm^–1^ ^42,44^ (Figure 3C, yellow and blue important bands on the left). This indicates that specific alterations in the backbone geometry of proteins lead to interactions with localized, distinctly organized membranes, when endolysosomes form at elevated ceramide level.

The endolysosomal remodeling patterns that are observed when treatment occurs with the higher inhibitor concentration of 10 μM (Scheme 1B and Figure 3D) differ from those observed under any of the other conditions. It leads to a co-occurrence of amide I components at 1707 cm^–1^ ^41^ and 1646 cm^–1^ ^28^ (Figure 3D, blue important band on the left) along with a tryptophan/tyrosine signal at 1616 cm^–1^,^43^ suggesting an altered conformation of proteins that are rich in hydrophobic residues. In agreement with this, different signals that can be associated with changes in the environments of disulfide bridges at 464 cm^–1^ ^42,44^ and 509 cm^–1^ ^11,28,42,44,46^ (Figure 3D, blue important bands on the left) are found as well. The amide III band at 1271 cm^–1^ ^43,49,56^ and the C–S band at 658 cm^–1^ ^11,42,44,46^ co-occur, further pointing to distinct interactions among proteins that underwent conformational changes. Interestingly, correlation of the bands at 1102 cm^–1^ ^50,51^ and 1439 cm^–1^ ^11,42–44^ is found, similar to the situation of lower inhibitor dose (compare Figures 3D with C).

A key hallmark when AC inhibition is induced at the higher SACLAC concentration (Figure 3B,D) is the distinct signal of adenine at 725 cm^–1^ and 730 cm^–1^,^28,43^ respectively (Figure 3C,D, red blocks in the maps). It co-occurs with an amide II component at 1500 cm^–1^ ^28^ and with the phospholipid C–C stretching mode at 1102 cm^–1^ ^50,51^ in the preformed endolysosomes (Scheme 1A, Figure 3C). In the RF analysis of the SACLAC-first experiments (Scheme 1B), it is highly associated with the band of tryptophan at 768 cm^–1^ ^28,43,63^ (Figure 3D, blue important band on the left). Although the cellular toxicity that we find for an incubation of the cells with 10 μM SACLAC is only moderate (Figure S2), we ascribe the presence of the adenine features to a changed degradation of nucleic acids in the endolysosomes, indicating a dysfunction of this compartment as a consequence of AC inhibition and concomitant ceramide accumulation. Such effects on endolysosomal function could be mediated by an enhanced lysosomal acidification that has been reported when AC was inhibited by 2-oleoylethanolamine in another cell line.^64^

### Interactions of Proteins, Nucleic Acids, and Lipids in Endolysosomes Formed at High Inhibitor Concentration as Highlighted by Tryptophan and Cholesterol Modes

The effects of an inhibition of AC by SACLAC on the composition and interaction of proteins are revealed by a number of relationships of individual spectral components of the broad SMD peaks centered at 1366 and 617 cm^–1^ (Figure S5D), assigned to vibrations of tryptophan and cholesterol, respectively (depicted as two striped rows in Figure 3D). Apart from the signal of a lipid terminal CH_3_ bending at 1375 cm^–1^ ^11^ (Figure 3E, yellow important band on the left), the features in the 1344–1380 cm^–1^ region (Figure 3E, blue important bands on the left) are attributed to tryptophan ring vibrations.^11,28,42–44^ The higher vibrational frequency of these bands, observed in these cells at 1354 cm^–1^ and 1348 cm^–1^ (Figure 3B), indicates that the tryptophan side chains must be involved in stronger hydrophobic interactions with surrounding molecules.^65^ The co-occurrence of vibrations of amide groups at 1702, 1645, 1587, and 1575 cm^–1^,^28,42^ of the protein backbone at 1140 cm^–1^,^42,44^ amino acid side chains at 1608 and 768 cm^–1^,^28,43,49,56,63^ disulfide bonds at 465 cm^–1^,^28,44^ and the lipid chain at 1120 cm^–1^ ^11,43,50^ (Figure 3E, blue and yellow blocks in the map) with the 1366 cm^–1^ tryptophan band (Figure 3E, blue important band on the left) indicates that proteins of different conformation and stability are present at the lipid membrane. In contrast, a signal of tryptophan at 1362 cm^–1^ (Figure 3E, blue important band on the left) is related to a signal of nucleic acids at 810 cm^–1^ ^43^ (Figure 3E red block in the map), indicating an interaction of proteins and nucleic acids. When the tryptophan signal is shifted to 1345 cm^–1^, only a lipid acyl chain vibration at 1120 cm^–1^ ^11,43,50^ and a DNA/RNA band at 825 cm^–1^ ^43^ co-occurred (Figure 3E), suggesting less hydrophobic interactions of tryptophan residues with nucleic acids next to lipid membranes.

Band co-occurrence within the 592–630 cm^–1^ region exhibits diverse patterns (Figure 3F). While an adenine signal at 729 cm^–1^ ^28,43^ (Figure 3F, red blocks in the map) and the tryptophan ring vibration at 773 cm^–1^ ^28,43,63^ (Figure 3F, blue blocks in the map) are related to the selected features at 617 and 621 cm^–1^ (Figure 3F, yellow important bands on the left),^41,43,59^ the feature at 617 cm^–1^ is moreover associated with a lipid CH_2_ scissoring mode at 1440 cm^–1^ ^11,42–44^ and a proline vibration at 906 cm^–1^ ^28,43^ (Figure 3F, yellow and blue blocks in the map). This is an indication that proteins and nucleic acids colocalize in specific, distinct membrane regions. Similar protein–nucleic acid interactions, but near cholesterol-rich membranes, are suggested by the related bands of the cholesterol features around 613 cm^–1^.^43^ For cholesterol band components shifted to lower frequencies, sequential co-occurrence patterns are observed, one at 608 cm^–1^ ^43^ with the amide II component at 1575 cm^–1^,^28,42^ one at 606 cm^–1^ ^43^ with amide II, a phenylalanine band at 1028 cm^–1^,^43,47–49^ a tyrosine vibration at 641 cm^–1^,^41,49,59^ and one at 603 cm^–1^ ^43^ with phenylalanine and tyrosine bands (Figure 3F and Table S1). The shift in important frequency that is accompanied by changes in band relations indicates a range of protein orientations on cholesterol domains, from the proximity of the amide backbone to exposure of residue side chains.

An inverse change was observed for phosphoinositide vibrations in the 595–599 cm^–1^ spectral range.^43^ With a shift of this mode from 599 cm^–1^ to 595 cm^–1^ (Figure 3F, blue important band on the left), a co-occurring tyrosine band at 641 cm^–1^ ^41,49,59^ (Figure 3F, blue block in the map) is replaced by a phenylalanine band at 1028 cm^–1^ ^43,47–49^ and a signal from protein backbones at 1139 cm^–1^ ^42,44^ (Figure 3F, blue blocks in the map). These relationships are indicative of an altered binding of proteins with phosphoinositides during ceramide enrichment.

### Distinct Protein–Lipid Interaction Network Is Observed upon ASM Addition

The SMD relation analysis also demonstrates differences of the molecular interaction network in response to an increase in ceramide when exogenous ASM was added to the cells (Scheme 1C, Figure 3G). The results show consistent co-occurrence of amide II components at 1572 cm^–1^,^28,42^ 1548 cm^–1^,^11,43,51,54^ and 1528 cm^–1^ ^28^ with the bands assigned to lipid chain deformation at 1319 cm^–1^,^28,30,42^ the protein backbone at 1132 cm^–1^,^42,44^ and disulfide bridges at 505 cm^–1^ ^11,28,42,44,46,47^ (Figure 3G, yellow and blue blocks in the map). This suggests that also upon ASM-related ceramide increase, conformational changes of membrane proteins contribute to the main biochemical differences. The cholesterol band at 609 cm^–1^ ^43^ (Figure 3G, yellow important band on the left) co-occurred with the amide II component at 1530 cm^–1^ ^28^ and the lipid CH_2_ twisting mode at 1302 cm^–1^ ^11,28,41,59^ (Figure 3G, blue and yellow blocks in the map). This indicates that the changed interactions of membrane proteins due to ceramide build-up occur in the cholesterol-rich domains. Interestingly, the lipid phosphate vibration at 1182 cm^–1^ ^11,30,42^ (Figure 3G, yellow important band on the left) is related with the protein amide II and backbone signals at 1516 cm^–1^ ^28^ and 1136 cm^–1^,^28,42,44^ respectively (Figure 3G, blue blocks in the map), implying changes of functional protein-stabilized phospholipid membranes. The interactions revealed in this analysis suggest that membrane-associated proteins may exhibit distinct structural disorder alongside changes in fluidity of the membranes surrounding them and changed dynamics of signaling lipids after ASM delivery, specifically due to the known enrichment in ceramide upon addition of ASM to the cells.^6^

### Exogenous Ceramide Has Indirect Effects on Endolysosomal Biochemistry

The effects of an increased ceramide concentration as a consequence of changes in sphingolipid metabolism were compared to those of exogenous ceramide that we added to the cell culture medium at four different concentrations. The range of ceramide concentration, from 1 to 15 μM, was chosen to enable a common incubation time and probing of large numbers of adhering cells in the absence of compromised appearance for all samples. At ceramide concentrations of 25 μM in the culture medium, cells detached visibly within short periods of time from the surface of the incubation wells (data not shown), in agreement with the dose-dependent decrease in viability described in previous work.^66,67^ The spectra of the cells incubated according to Scheme 1D share a number of similarities that are independent of ceramide dose (cf. Figure S6 and discussion of the frequency of occurrence of spectral bands in the Supporting Information). In line with our prior studies using labeled ceramide,^30^ the results indicate that despite its primary localization in the Golgi,^68^ ceramide that is taken up from the environment into the cells induces changes in the molecular environment of endolysosomes that are independent of ceramide concentration.

Nevertheless, the SERS data from the endolysosomes of cells that took up additional ceramide from the culture medium also display changes with respect to their control group that vary with ceramide dose, as indicated by an RF analysis, achieving an overall accuracy of 84.65% (Table S3). An increasing sensitivity for the separation with increase in ceramide dose is observed (Table S3) and implies that specific molecular structure and interactions represented by respective spectral features become more discriminative at higher ceramide concentrations. They concern particular influences on protein stability as well as changes in the structure, composition, and interaction of lipid membranes and nucleic acids (cf. Figure S6 and corresponding discussion). Accordingly, the important bands selected by SMD (cf. Figure S8) are attributed to vibrations of proteins and lipids and exhibit diverse correlations with other selected bands (cf., Figure S4).

**Figure 4:**
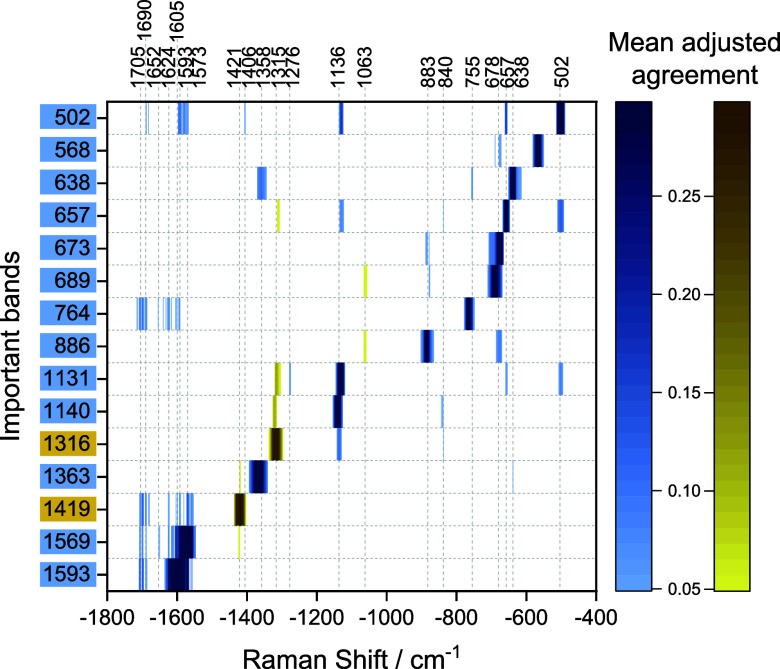
Spectral variables co-occurring with the important bands (left) selected by SMD analysis (cf. Figure S8) of SERS spectra from 3T3 cells incubated with ceramide at different concentrations after incubation with gold nanoparticles for 24 h. Bands assigned to proteins are marked in blue and to lipids in yellow.

The bands at 1594 cm^–1^ and 1570 cm^–1^ assigned to phenylalanine^43,49,56^ and amide II,^28,42^ respectively, co-occur with each other as well as with the amide I bands at ∼1690 cm^–1^ (Figure 4, blue blocks in the map),^43^ suggesting changes of secondary structure and side chain exposure. The bands of amide groups co-occur with a lipid marker at 1420 cm^–1^ (Figure 4, yellow important band on the left).^28,42,57^ Moreover, the relations between a lipid band at 1315 cm^–1^,^30,42^ the amide III component at 1276 cm^–1^,^28,42,43^ disulfide bands at 657 cm^–1^ and 500 cm^–1^,^11,28,42,44,46,47^ and the protein backbone vibration at 1131 cm^–1^ ^42,44^ (Figure 4, yellow and blue blocks in the map) indicates coordinated changes of protein structures and the organization of surrounding membranes induced by the exogenous ceramide. The C–S stretching vibration at 689 cm^–1^ ^56^ (Figure 4, blue important band on the left) co-occurs with the lipid acyl chain stretching at 1061 cm^–1^ ^43,47,50,51,56^ as well as the proline/valine at vibration at 878 cm^–1^ (Figure 4, yellow and blue blocks in the map).^28^ This spatial colocalization of these signals of methionine and proline/valine and bands assigned to hydrophobic membrane regions indicate that the changes induced by supplemented ceramide affect methionine and aliphatic amino acids differently.

The tryptophan residue interactions demonstrated distinct microenvironments upon exogenous ceramide treatment, as indicated by the relation of the tryptophan vibration at 1363 cm^–1^ ^43^ with reorganized lipids and cysteine residues (Figure 4, blue important band on the left), evidenced by co-occurring bands at 1421 cm^–1^ ^28,42,57^ and 638 cm^–1^,^41,49,59^ respectively (Figure 4, yellow and blue blocks in the map). The ring breathing vibration at 764 cm^–1^ ^28,43,63^ (Figure 4, blue important band on the left) is associated with amide I at 1705, 1690, and 1652 cm^–1^,^28,41^ as well as aromatic amino acid vibrations at 1624 ^43^ and 1605 cm^–1^ ^43,49,56^ (Figure 4, blue blocks in the map), suggesting alterations in the local environment of hydrophobic residues accompanied by partial rearrangement of protein secondary structures. Another important tryptophan feature at 568 cm^–1^ ^43^ (Figure 4, blue important on the left), which is related to a shifted C–S bond at 678 cm^–1^ ^41^ (Figure 4, blue block in the map), may indicate changes in the spatial arrangement between tryptophan and sulfur-containing amino acids.

The results from our SMD analysis demonstrate the tight coordination between protein conformational changes and membrane remodeling processes in the endolysosomes when ceramide is supplemented. These results confirm that exogenous ceramide has indirect effects on membrane proteins and associated membranes in the endolysosomes, despite the known translocation of exogenous ceramide to the Golgi apparatus rather than to lysosomes.^68^ The observation is in good agreement with the results of our previous work that showed that fluorophore-labeled ceramide affected the molecular interactions in endolysosomes indirectly.^30^ Furthermore, JEG3 trophoblastic cells that had been treated with exogenous C16:0 ceramide showed lysosomal alterations, including increased acidification and volume.^64^

### Endolysosomal Alterations Are Specific of Timing and Origin of Ceramide Stress

The analysis demonstrates how endolysosomes respond to ceramide enrichment induced by (i) blocked degradation, (ii) enzymatic production, and (iii) exogenous supply. All conditions induce the reorganization of membrane proteins and endolysosomal membranes, evident by many different alterations of protein structure and protein–lipid interactions. However, the dose, timing, and origin of ceramide stress result in distinct effects on composition, structure, and interaction of these molecules.

When higher concentrations of the AC inhibitor SACLAC were applied (Figure 3B,D–F), endolysosomes display structural features that can be related to dysfunction, indicating that a stronger inhibition of AC could exceed the capacity of the cells to maintain endolysosomal homeostasis by adaptation in composition. It was shown that AC inhibition by SACLAC can lead to accumulation of ceramide with long fatty chains in different acute myeloid leukemia cells,^10^ including C16 ceramide species that are most often pro-apoptotic.^69^ When SACLAC prevents the breakdown of ceramide to sphingosine, a consequent decrease was also observed for the further metabolic product sphingosine-1-phosphate (S1P),^10^ a pro-survival lipid,^70^ that together with ceramide constitutes a crucial sphingolipid rheostat to determine cellular death or proliferation.^70,71^ The addition of SACLAC in a high concentration likely disturbs the balance between ceramide and S1P in the endolysosomes, thereby leading to more complex molecular changes. The spectral changes in phospholipid composition and packing are likely a consequence of altered lipid composition, caused, e.g., by depletion of S1P by the action of SACLAC.

In the experiments where endolysosomes containing nanoprobes had formed before the inhibitor SACLAC could act on the AC enzyme (Scheme 1A), more signatures of destabilized protein structures were found (Figure 3A,B). We conclude from this that the attempt of the cell to buffer the acute increase of ceramide involves major modifications in protein structure and composition. In contrast, exposure to the inhibitor before optical nanoprobes incubation (Scheme 1B), i.e., probing of endolysosomes that form during the ongoing enzyme inhibition, yields more spectral features related to protein–lipid interfaces (Figure 3C–F). In this situation, a process of metabolic adaptation has already been taking place, and different requirements or consequences in endolysosome formation include the organization of proteins at the endolysosomal membrane of altered lipid composition and structure. Such differences reflect the sensitivity of the endolysosomal environment to the timing of interactions between the affected molecules and the probing nanoparticles.

Compared to AC inhibition, direct ceramide generation via additional ASM in the endolysosomes clearly indicates changes of protein and lipid stabilization evidenced by signals from the protein backbones and lipid composition and structure (Figure 3G). In addition to a known rapid ceramide production,^58,61^ the decrease in sphingomyelin due to its enhanced consumption may lead to changes in membrane properties and function, including the association with membrane proteins.^72^ The observed spectral changes of cholesterol and phospholipids (seen in Figure 3) are in line with different lipid profiles that are a consequence of the depletion of sphingomyelin by increased ASM activity on the one hand and on the other hand a necessity to maintain endolysosomal homeostasis in the presence of excessive ceramide levels.

In contrast to the accumulation of ceramide in situ by modified enzyme action, the spectral differences observed in the cells exposed to exogenous ceramide (Figure 4) reflect an endolysosomal response to actual systemic cell stress. Exogenous ceramide is metabolized to sphingomyelin and glucosylceramide in the Golgi apparatus,^73^ before it is transferred to the plasma membrane via vesicular transport pathways or lipid-transfer proteins.^74^ The adaptation of the Golgi apparatus and plasma membrane to increased lipid levels may lead to changes in endolysosomal lipid composition and different protein–membrane interactions through lipid traffic between compartments. As a pro-apoptotic agent, C16-ceramide was also shown to induce cytotoxicity in adenocarcinoma HCT116 cells partially via apoptotic processes, indicated by increased caspase-3 activity and poly­(ADP-ribose) polymerase cleavage.^67^

### Ceramide Stress Changes the Endosomal and Mitochondrial Ultrastructure and Processing of Gold Nanoprobes

The ultrastructures of the manipulated cells and the distribution of the optical nanoprobes were characterized by soft X-ray nanotomography (SXT).

Upon inhibition of AC by SACLAC, some vesicular structures are observed to have lost their typical spherical shape and instead display angular outlines in the tomographic slices (blue asterisks in Figures 5A–C, and S9A,C). In the cells exposed to SACLAC at 10 μM (Figures 5B and S9B,C), ASM (Figures 5D and S9D), and ceramide at 5 μM (Figure 5E), several vesicles show significant heterogeneity and high complexity, indicated by red markings. The abnormal shapes and multivesicular structures likely represent endolysosomes undergoing membrane reorganization and morphological alteration due to an overload of ceramide.^75–77^ Ceramide can form distinct microdomains in phospholipid membranes through strong headgroup interactions, leading to significant changes in membrane organization.^77^ These properties affect the ultrastructure and interaction of ceramide containing vesicles outside and inside cells. For example, ceramide generated by enzyme application can trigger vectorial vesicle formation including endocytosis and budding in giant liposomes composed of phosphatidylcholine and sphingomyelin.^76^ Such changes at the ultrastructural level were shown to have a direct influence on the biological function of the affected cells and tissues. In mouse fibroblast cells, exogenously added C6-ceramide was found to induce rapid and reversible endocytic vesicle formation,^78^ and fibroblasts from Farber disease (AC activity deficient) patients were reported to contain swollen lysosomes with lamellar and curvilinear membrane inclusions.^7^

**Figure 5:**
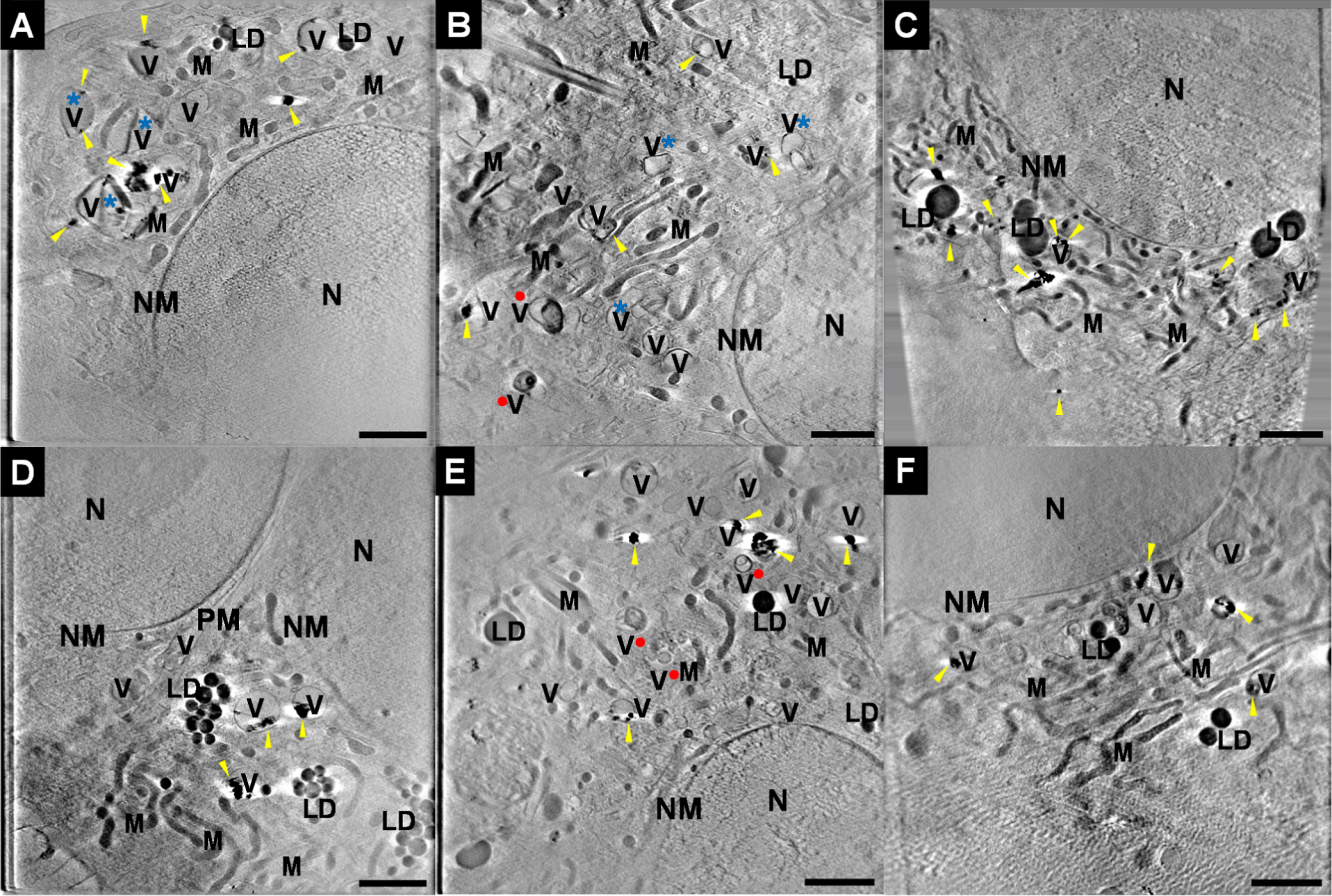
Reconstructed X-ray tomograms of 3T3 cells incubated with (A) 1 μM SACLAC and (B) 10 μM SACLAC for 24 h after incubation with gold nanoparticles for 24 h (cf. Scheme 1A), (C) with gold nanoparticles for 24 h after incubation in the culture medium with 10 μM SACLAC for 24 h (cf. Scheme 1B), with (D) 200 nM ASM for 3 h (cf. Scheme 1C) and (E) 5 μM ceramide for 15 min (cf. Scheme 1D) after incubation with gold nanoparticles for 24 h, and (F) with gold nanoparticles for 24 h as the control sample. Additional tomograms of the cells exposed to SACLAC and ASM are shown in Figure S9. M: mitochondrion, V: vesicle, NM: nuclear membrane, N: nucleus, LD: lipid droplets, ER: endoplasmic reticulum. Gold nanostructures are marked with yellow arrowheads. Red dots and blue asterisks mark the affected vesicles. Scale bar: 2 μm.

The drastic changes in endolysosome ultrastructure are in agreement with changes of lipid composition and organization exhibited by the spectral data and together with them underpin the disruption of lipid metabolic balance by SACLAC, ASM, and exogenous ceramide.

Elongated mitochondrial networks, a small number of condensed, dark structures, and swollen structures with visible cristae were observed in all treated cells (Figures 5A–E, S9A–D) but not in the controls (Figure 5F). This suggests that the mitochondria undergo mild stress due to the affected endolysosomal metabolism and is in agreement with the fragmentation and conformational changes of membrane proteins that is revealed by the corresponding spectral features and their relations (Figures 3 and 4). Future work will use mitochondria-targeted SERS probes to explore vibrational changes upon lipid regulation in the mitochondrial compartment as well.

The processing of the gold nanoparticles that were used as optical nanoprobes in the SERS experiments and the properties of their intraendolysosomal agglomerates were also analyzed, as they provide information on an endolysosomal ultrastructure, as well. The gold nanoparticles form loose aggregates of irregular shapes in the vesicular structures throughout endocytic processing, in agreement with our previous findings in this and other cell lines.^30,44,79^ The sizes of the nanoaggregates vary according to the distinct interference with ceramide metabolism. When exposure to the AC inhibitor SACLAC followed the endocytosis of gold nanoprobes (Scheme 1A), larger agglomerates were observed in a size range of around 40–840 nm (Figures 5A and S9A), similar to the maximum size of about 500 nm in the control samples (Figure 5F). With higher SACLAC concentration, small aggregates and/or individual particles were observed with significantly higher frequency (Figures 5B and S9B). The latter observation supports the conclusions drawn from the spectral data that contributions to the spectra from adenine and destabilized proteins (Figure 3B) likely result from the vesicular destabilization observed here. The possibility of vesicle disintegration as a consequence of stronger ceramidase inhibition is supported by work that reported leakage of vesicle content after ceramide accumulation in phospholipid membranes.^80^

When the cells were first exposed to SACLAC at high concentration and only later to the nanoparticles (Scheme 1B), a higher number of aggregates were found in the size range of 100–300 nm. Some aggregates even reach sizes of up to 1200 nm (Figures 5C and S9C). In this condition, the cell membrane composition and endolysosomal environment, which has already adapted to high ceramide content, likely promotes the uptake of gold nanoparticles and has an influence on the endosomal fusion and formation of larger structures. An extensive uptake of nanoprobes and their aggregates agrees with a broader set of endolysosomal changes captured in the SERS data, as determined by SMD analysis (Figure 3D).

The size of the intracellular agglomerates of the gold nanoparticles also increases when ceramide content is increased by additional ASM (Figure 5D) and exogenous ceramide (Figure 5E), reaching a maximum of around 950 and 820 nm, respectively. However, neither the presence of small aggregates indicative of vesicle disintegration nor altered geometric shapes of endolysosomal vesicles were found upon these treatments, unlike in the cells incubated with SACLAC (Figures 5A–C and S9A–C). Degradation of both ceramide and ASM can play a role here. Ceramide can be converted into sphingosine, glucosylceramide, and other sphingolipids.^4^ Like other proteins targeted to the lysosome, recombinant ASM would be eventually degraded by other lysosomal enzymes such as cathepsins.^61,81^ Compared to the irreversible inhibition of AC when the cells are exposed to SACLAC for 24 h,^40^ the short-term exposure to ASM for 3 h and the Golgi-targeted exogenous ceramide for 15 min may not manifests in detectable changes to the ultrastructure, despite the changes in molecular composition and structure detected in the endolysosomes by optical nanospectroscopy.

## Conclusions

We analyzed the changes in molecular structure and interactions in the endolysosomal microenvironment of fibroblast cells with elevated ceramide content based on SERS spectra in combination with 3D ultrastructural information from nanotomography. As indicated by the vibrational bands and their relations, elevated ceramide levels, generated by (i) the inhibition of AC inhibitor SACLAC, (ii) increased synthesis of ceramide with supplemented recombinant ASM, and (iii) exogenous ceramide, have distinct biochemical effects. Our data show that the regulation of ceramide as necessary response via known metabolic pathways is accompanied by specific endolysosomal molecular changes, also when cytotoxicity is low or absent. Several changes in the molecular makeup in the endolysosomal environment that include the stability of membrane structures, protein composition and structure, and protein–lipid interactions are supported by the morphological constitution of this compartment and its processing of the optical gold nanoprobes, as well as in the ultrastructure of mitochondria, as revealed by nanotomography.

The biochemical response to ceramide stress in the endolysosomes displays diverse spectral signatures dependent on the dose, enzymatic pathway, and origin of built-up ceramide. The induction of ceramide stress as a consequence of the modulated enzyme activity and addition of external ceramide, respectively, was found to affect the protein–lipid interactions and is likely connected to the structure and function of the endolysosomes. These indirect changes in this compartment have so far not been captured by state-of-the-art optical microscopy, using conventional labeling methods.

The spectral data indicate that the onset of AC inhibition relative to the formation of the probed endolysosomes determines how the stability and interactions of proteins with lipids are affected by high ceramide levels. Changes in the size and stability of endolysosomes that form during ongoing inhibition of AC observed by nanotomography support this observation. The relation of spectral differences indicates that higher AC inhibitor concentrations disturb the endolysosomal homeostasis to a greater extent, as indicated by dysfunction-related vibrational markers.

Promoting sphingomyelin hydrolysis by additional ASM yields ceramide enrichment as well, yet the resulting differences in protein and lipid stabilization show other specifics, particularly changed interactions of membrane proteins in the cholesterol-rich membrane regions. In contrast, the addition of external ceramide was found to impact the endolysosomal conditions indirectly via its involvement in lipid metabolic processing and trafficking processes from the Golgi and induced subtle interaction changes of proteins and nearby membranes in a dose-dependent manner. The identification of such small dose-dependent effects was achieved by a comparison of the molecular effects in situ in the cells under slightly varied experimental conditions.

The detailed information on a specific molecular structure and interaction illustrates the methodological advantage of the applied data analysis approach, which relies on SMD and the utilization of relationships of characteristic spectral features. Together with the highly localized collection of the SERS data from the endolysosomal volumes, the indicated relationships and/or co-occurrences of signals of particular functional groups or molecules allow us to identify weak, complex interactions between colocalized biomolecules in living cells. In future work, probing of other cellular compartments, such as mitochondria, by this approach will provide further insights into the activity of these and other enzymes, possibilities for their manipulation, and a better understanding of the effects thereof at the subcellular level.

## Experimental Section

### Preparation of Gold Nanoparticles, SACLAC, and ASM

Gold­(III) chloride hydrate, sodium citrate dehydrate, and ceramide were purchased from Sigma-Aldrich (Steinheim, Germany). Citrate-stabilized gold nanoparticles (∼30 nm) were synthesized following the protocol in ref 82 using solutions prepared with Milli-Q ultrapure water. SACLAC was synthesized as described in ref 40 ASM was expressed in insect Sf9 cells and purified to homogeneity following the protocol described in ref 83.

### Cell Culture and Sample Preparation

The Swiss albino mouse fibroblast cell line 3T3 (DSMZ, Braunschweig, Germany) was cultured in Dulbecco’s Modified Eagle’s Medium (DMEM, Bio&SELL, Nürnberg, Germany) containing 10% fetal calf serum (FCS, Biochrom, Berlin, Germany) at 37 °C with 5% CO_2_.

All cells prepared for SERS experiments were grown on glass coverslips. For SERS experiments of SACLAC treatment after nanoprobe incubation (Scheme 1A), ASM treatment (Scheme 1C), and incubation with exogenous ceramide (Scheme 1D), the cells were grown on glass coverslips for 24 h. Then, the culture medium was exchanged with a dilution of gold nanoparticles in DMEM-FCS of 1:10 (nanoparticle concentration ∼10–11 M) for 24 h. The cells were rinsed with phosphate-buffered saline (PBS) (Bio&SELL, Nürnberg, Germany) to remove excess gold nanoparticles. For SACLAC treatment and ASM treatment, the cells were incubated at 37 °C in DMEM-FCS with 1 μM and 10 μM SACLAC for 24 h and with 200 nM ASM for 3 h, respectively. Control samples corresponding to Scheme 1A,C were incubated at 37 °C in fresh DMEM-FCS for 24 and 3 h, respectively, after the incubation with gold nanoparticles for 24 h.

For incubation with exogenous ceramide, the culture medium was exchanged with DMEM-FCS containing 1 μM, 5 μM, 10 μM, and 15 μM ceramide. Cells were incubated at 4 °C for 30 min, then rinsed with PBS, and grown in fresh DMEM-FCS at 37 °C for 15 min as proposed previously.^68^ Control samples corresponding to Scheme 1D were incubated at 4 °C for 30 min and then at 37 °C for 15 min in fresh DMEM-FCS, after incubation with gold nanoparticles for 24 h.

For incubation with the gold nanoprobes in cells that were pretreated with SACLAC (Scheme 1B), cells were grown with 1 and 10 μM SACLAC in DMEM-FCS at 37 °C for 24 h. Then, the culture medium was exchanged with a dilution of gold nanoparticles in DMEM-FCS of 1:10 (nanoparticle concentration ∼10–11 M) for 24 h. Control samples were grown in DMEM-FCS at 37 °C for 24 h and then incubated with gold nanoparticles for 24 h.

All cell samples were prepared across two independent cell passages (biological replicates). For each condition, cells were cultured in the three wells of 6-well plates. SERS experiments on the cells were collected from a minimum of eight randomly selected cells in PBS after rinsing with PBS three times, resulting in over 3200 spectra per condition.

To prepare the samples for cryo soft X-ray nanotomography (SXT), cells were grown on Formvar-coated gold grids (Quantifoil, Jena, Germany) and incubated as described above. After the incubation, each grid was rinsed three times with PBS, the excess buffer was blotted with filter paper, and the grids were plunge-frozen in liquid ethane.

### SERS Experiments

Raman spectra of the cytoplasm of cells were obtained in a microspectroscopic setup by excitation with a 785 nm diode laser (Toptica, Munich, Germany) by using a single-stage spectrograph equipped with a CCD detector (Horiba, Munich, Germany). A 60× water immersion objective was applied, resulting in an intensity of 2.7 × 10^5^ W/cm^2^ at the sample. The focal volume was ∼10 fL. All spectra were recorded with an acquisition time of 1 s and at a spectral resolution of ∼2 cm^–1^, considering the full spectral range used from 300 to 1900 cm^–1^.

### Cryo Soft X-ray Tomography

A transmission X-ray microscope equipped with a cryostage at beamline U41-PGM1-XM at the electron storage ring BESSY II (Helmholtz-Zentrum Berlin für Materialien und Energie, Berlin, Germany) was employed to examine vitrified cell monolayers with a thickness of approximately 10 μm.^84^ For each individual cell, tilt series composed of up to 130 images were acquired in 1° steps across multiple angular ranges with a pixel resolution of 9.8 or 6.9 nm (25 nm zone plate objective) at a 510 eV photon energy condition. Depending on the sample thickness, the exposure time was adjusted for each tilt angle series between 1 and 4 s per image. With intracellular gold nanoparticles as fiducial markers, corrected tilt series were reconstructed into tomograms with Etomo software (IMOD, Colorado, USA), using the back-projection or simultaneous iterative reconstruction technique.

### Data Analysis

The SERS spectra were frequency-calibrated using a toluene–acetonitrile mixture (1:1) standard and preprocessed in MatLab R2020b (The MathWorks, Inc., Natick, MA, USA) including spike removal, baseline correction using asymmetric least-squares,^85^ and vector-normalization. Only the spectra with detectable signals were kept for further analyses, using the full spectral range from 400 to 1800 cm^–1^. Average spectra of each condition were calculated for a further qualitative analysis of the SERS signals. Relative occurrences of signals were evaluated in Mathematica 12.1 (Wolfram, Champaign, IL, USA) following a method previously reported.^44^ Briefly, the integrals of the spectra were calculated by moving an integral window selected here to be ±10 cm^–1^ around a central wavenumber, with predefined steps between central wavenumbers, chosen here to amount to 5 cm^–1^. If the integral at a central wavenumber exceeds the average integral in a signal-free reference spectrum by a defined threshold, then a signal is summed up in the given central wavenumber bin. This was performed at every central wavenumber for each individual spectrum, yielding a histogram. PC analysis (PCA) was performed with the preprocessed individual SERS spectra in MatLab R2020b.

RF and SMD analyses were conducted in R 4.2.2 ^86^ using *ranger* 0.15.1 ^87^ and *RFSurrogates* 0.4.2,^32,88^ respectively. For each comparison, an RF classification model was trained to differentiate between the spectra of the respective conditions and the control samples. This model was trained using 80% of the spectra that were randomly selected, and the remaining 20% of the spectra were used as test data. For training, the number of trees (*num.trees*) and the number of variables to consider in each split (*mtry*) were set to 10′000 and 161 (corresponding to *p* ^3/4^, where *p* is the total number of spectral variables), respectively. SMD was performed by using the RF parameters defined above and a predefined number of surrogate splits (*s*) of 88, corresponding to 0.10 *p*. Important variables were selected based on the SMD importance score with lower value indicating greater importance. Consequently, variables with SMD values below the defined threshold were selected and the MAA for pairwise relationships of each spectral variable was determined. For clarity, the variable with the lowest SMD importance value served as representative for one signal. Their MAA values to all *p* spectral variables were depicted to visualize the relation between the important signals and spectral variables characteristic of specific molecules.

## Supplementary Material



## Notes

Spectral data from control samples in the experiments with cells treated with SACLAC; viability of cells after treatment with SACLAC; PC analysis of spectra from cells treated with SACLAC; PC analysis of spectra from cells exposed to additional ASM; RF-based analysis of SERS spectra from 3T3 cells treated with SACLAC and ASM; SERS data of cells incubated with ceramide at different concentrations; PC analysis of SERS spectra from cells treated with ceramide; RF-based analysis of SERS spectra from 3T3 cells treated with ceramide; and soft X-ray tomograms of 3T3 cells treated with SACLAC and ASM (PDF)

## #

Chemistry Department, Faculty of Science, Suez Canal University, Ismailia 41522, Egypt

## Abbreviations

ASM: acid sphingomyelinase
CER: ceramide
PCA: principal component analysis
RF: random forest
SACLAC: N-[(2*S*,3*R*)-1,3-dihydroxyoctadecan-2-yl]­2-chloroacetamide
SERS: surface-enhanced Raman scattering
SMD: surrogate minimal depth
