# Supplementary material for: Endolysosomal Impact of Elevated Ceramide Levels Revealed by Optical and Ultrastructural Nanoprobing
Source: ACS Nano. 2026 May 4;20(19):14071–87. doi: 10.1021/acsnano.5c22213 (PMC13192315; doi:10.1021/acsnano.5c22213)
Supplement: Supplementary file 1 [file nn5c22213_si_001.pdf]

Supporting Information

Endolysosomal Impact of Elevated Ceramide Levels Revealed by Optical and Ultrastructural Nanoprobing

Yiqing Feng[‡#], *Florian Gärber*[§], *Essa M. Saied*[‡※], *Harshita Singh*[‡], *Cecilia Spedalieri*[‡], *Stephan Werner*[w], *Christoph Pratsch*[w], *Christoph Arenz*[‡], *Stephan Seifert*[§], *Janina Kneipp*[‡*]

[‡] Department of Chemistry, Humboldt-Universität zu Berlin, Brook-Taylor-Str. 2, 12489 Berlin, Germany

[#] Einstein Center of Catalysis (EC2/BIG-NSE), Technische Universität Berlin, Marchstr. 6-8, 10587 Berlin, Germany

[§] Hamburg School of Food Science, Department of Chemistry, Universität Hamburg, Grindelallee 117, 20146 Hamburg, Germany

[※]Chemistry Department, Faculty of Science, Suez Canal University, Ismailia 41522, Egypt

[w] Helmholtz-Zentrum Berlin für Materialien und Energie GmbH, Department X-ray Microscopy, Albert-Einstein-Str. 15, 12489 Berlin, Germany

Contents

Spectral data from control samples in the experiments with cells treated with SACLAC S2
Viability of cells after treatment with SACLAC........ S4
Principal component analysis of spectra from cells treated with SACLAC S5
Principal component analysis of spectra from cells exposed to additional ASM S8
Random Forest based analysis of SERS spectra from 3T3 cells treated with SACLAC and ASM S9
SERS data of cells incubated with ceramide at different concentrations S12
Principal component analysis of SERS spectra from cells treated with ceramide S15
Random Forest based analysis of SERS spectra from 3T3 cells treated with ceramide........ S17
Soft X-ray tomograms of 3T3 cells treated with SACLAC and ASM S19

Spectral data from control samples in the experiments with cells treated with SACLAC

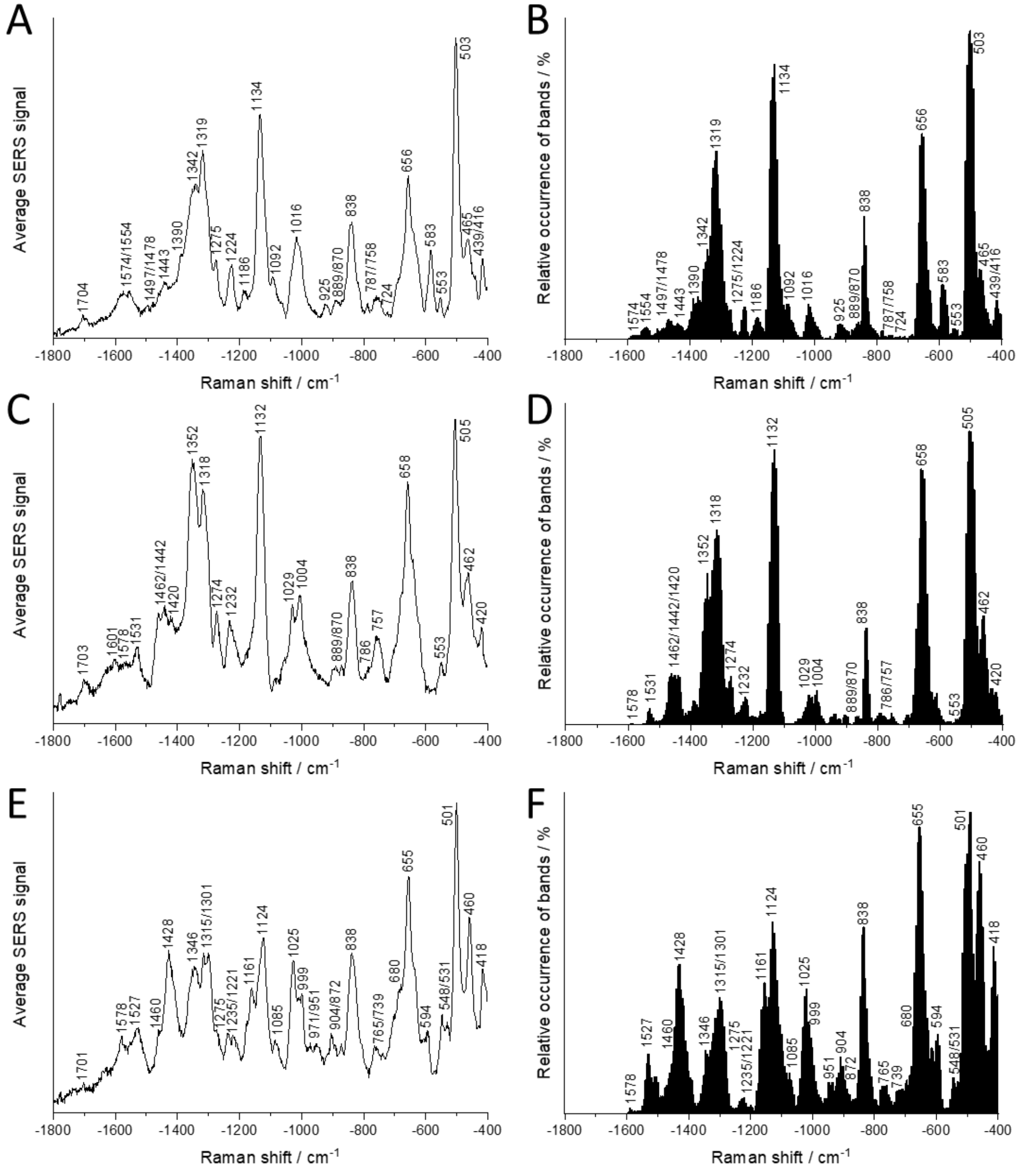

Figure S1. Average SERS spectra (A, C, E) and relative band occurrence (B, D, F) in the respective SERS data sets of 3T3 cells incubated with gold nanoparticles for 24 h as the control samples for cells incubated **(A, B)** with 1 µM SACLAC after gold nanoparticle incubation according to Scheme 1A, **(C, D)** with 10 µM SACLAC after gold nanoparticle incubation according to Scheme 1A and **(E, F)** with SACLAC prior to gold nanoparticles according to Scheme 1B. The data sets contain 687 (A, B), 451 (C, D) and 741 (E, F) SERS spectra, respectively, after elimination of spectra with no signal. Excitation wavelength: 785 nm. Excitation intensity: 2.7×10^5 W cm^{-2}. Acquisition time: 1 s.

Table S1. Raman shifts and tentative assignments of bands in the SERS spectra from cells. Assignments based on Ref 1-20 [a,b]

Raman Shift / cm^{-1}	Tentative assignment	Raman Shift / cm^{-1}	Tentative assignment
1747	Lipid C=O/C=C	1041	Tyr R def
1724	Lipid ester group	1028	Phe C-H ip bend
1703	Amide I, Asp, Glu COOH def	1008	Phe R br
1685	Amide I	998	
1646		971	Pro, Val C-C str
1620	Tyr/Trp C=C	949	
1606	Tyr, Phe R str, Amide I	926	
1599	Phe C=C def	915	Pro C-C str
1578	Amide II, C-C str, COO– str	901	Trp C-C def
1555	Amide II, Trp, Tyr, COO– str	890	Pro, Val C-C str
1539	Amide II	881	Trp R def
1529		872	Pro, Val C-C str, Pro R br
1519		845	Tyr R br
1497		837	
1476	Lipid CH_2 def	825	DNA/RNA phosphate ester
1462		810	
1443		802	Lipid O–P–O str
1434		789	DNA/RNA O-P-O str
1426		778	Phosphatidylinositol
1419	Amino acids COO– str	765	Trp R def
1411	Trp COO- str	755	Trp R br
1402	Amino acids COO– str	741	
1389	Lipid CH_3 bend	728	DNA, A
1378	Lipid CH_3 def	707	Cholesterol R def
1369	Trp	698	C–S str
1347		679	
1318	Lipid CH_2/CH_3 def	658	
1303	Lipid CH_2 def	643	Tyr C-C twist
1276	Amide III	625	Phe C-C twist
1257		613	cholesterol
1232		605	
1223	Amide III (β sheet structure)	595	Phosphatidylinositol
1213		586	S-S str
1207	Tyr, Trp, Phe	570	Trp
1180	Lipid phosphate ester	553	S-S str
1172	Tyr C-H bend	532	Cholesterol
1161	Protein C-N/C-C str	517	Phosphatidylinositol
1132		524	S-S str
1125		503	
1115	Lipid C-C str	460	
1100		439	Cholesterol
1088		426	
1063		417	Trp

[a] Str, stretching; def, deformation; twist, twisting; br, breathing; bend, bending; ip, in-plane; R, ring; Trp, tryptophan; Tyr, tyrosine; Phe, phenylalanine; Pro, proline; Val, valine; Asp, asparagine; Glu, glutamine; A, adenine

[b] Band positions of the same vibration mode can vary for different samples, in agreement with the variation of typical SERS spectra[21]

Viability of cells after treatment with SACLAC

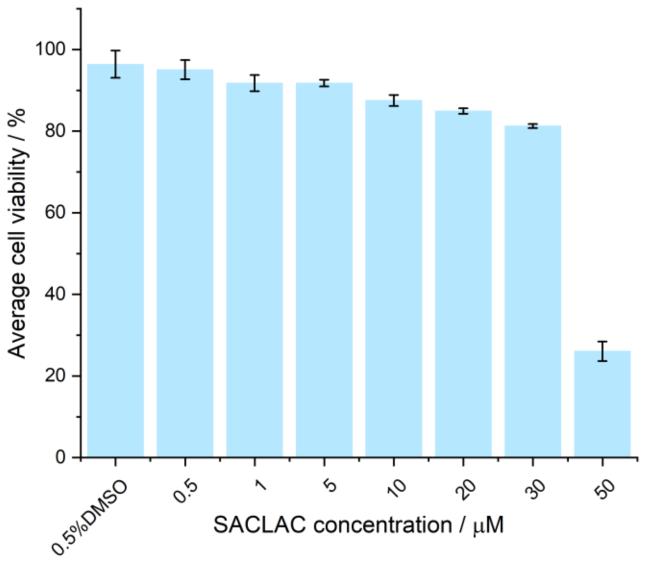

Figure S2. Cell viability (in %) of 3T3 cells after the incubation with SACLAC at different concentrations as indicated for 24 h.

The cell viability changes under SACLAC treatments were studied by XTT cell proliferation assay kit (Cayman Chemical, Michigan, USA). 3T3 cells were seeded in flat-bottom 96-well plates at a density of around 5000 cells per well in Dulbecco's Modified Eagle Medium (DMEM, Bio&SELL, Nürnberg, Germany) containing 10% fetal calf serum (FCS, Biochrom, Berlin, Germany) and cultured at 37°C with 5% CO_2. After 24 h, the medium was replaced with fresh medium containing varying concentrations of SACLAC (0.5 µM, 1 µM, 5 µM, 10 µM, 20 µM, 3 µM, 50 µM). As controls, cells were grown in the medium with 0.5% dimethyl sulfoxide (DMSO, vehicle control) and pure culture medium (negative control). Following a 24 h incubation with the enzyme inhibitor SACLAC, 10 µL XTT mixture was used according to the protocol from the manufacturer (Biozol Diagnostica, Eching, Germany). Medium without cells served as blanks to correct for background absorbance. The absorbance was measured at 450 nm using a microplate reader (Perkin-Elmer, Berlin, Germany). Cell viability (%) of each condition was calculated from 4 independent replicates. As Figure S2 shows, the SACLAC treatments at 1 and 10 µM reduced the viability to 91% and 87%, respectively.

Principal component analysis of spectra from cells treated with SACLAC

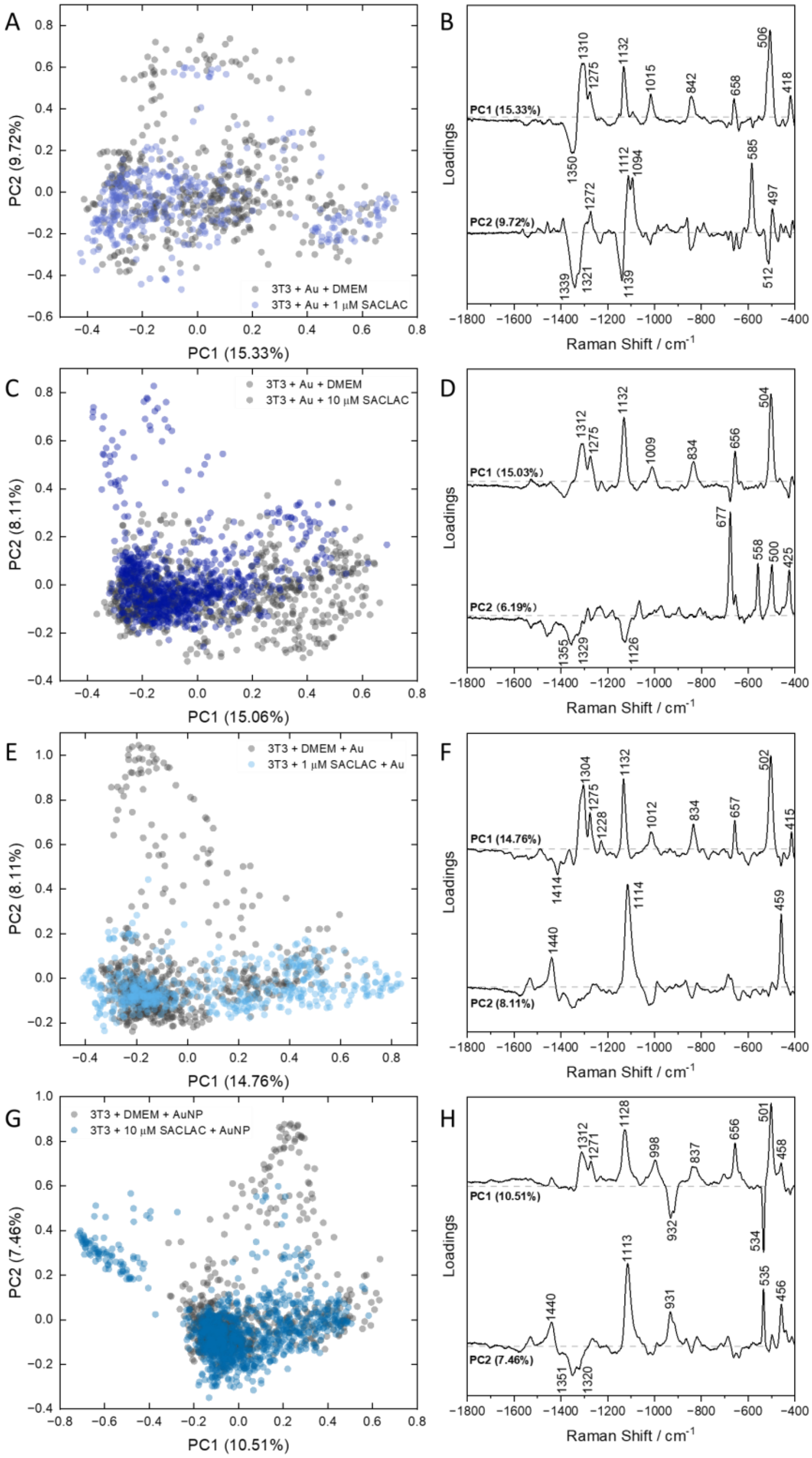

Figure S3. Results of the principal component analysis (PCA) applied to data sets of 3T3 cells treated with **(A, B)** 1 μM SACLAC, **(C, D)** 10 μM SACLAC for 24 h after incubation with gold nanoaprticles for 24 h, with **(E, F)** 1 μM SACLAC, **(G, H)** 10 μM SACLAC for 24 h prior to incubation with gold

nanoparticles for 24 h and the respective control groups. The analysis used the full spectral from 400 cm -1800 cm^{-1}. Scores (A, C, E and G) and loadings of PC1 and PC2 (B, D, F and H). are shown.

To evaluate the major spectral variation associated with ceramide accumulation due to acid ceramidase inhibition by SACLAC, we performed principal component analysis (PCA) on SERS data sets of the inhibitor treated cells and their respective controls. Figure S3 shows the score plots (Figure S3A, S3C, S3E, S3G) and loadings (Figure S3B, S3D, S3F, S3H) of the first two principal components (PC1 and PC2) of the PCA analysis of the data from cells treated with two SACLAC concentrations and the two incubation conditions.

The loading plots reveal both shared features, as well as differences that are specific to the molecular interaction changes induced by acid ceramidase inhibition. The loadings of PC1 (upper traces in Figure S3B, S3D, S3F and S3H) show consistent key spectral features from proteins and lipids between SACLAC treatment and control samples under different conditions. These include a lipid CH_2/CH_3 deformation mode at 1312 cm^{-1},[11, 18] an amide III signal at 1275 cm^{-1},[4, 11, 13] protein backbone vibrations at 1132 cm^{-1},[7, 11] disulfide C-S and S-S bands at 655 cm^{-1} and 500 cm^{-1},[2, 7, 11, 13, 17] respectively, and characteristic signals of phenylalanine at 1010 cm^{-1},[4] of tyrosine at 834 cm^{-1},[4, 6, 8, 17] and of tryptophan at 415 cm^{-1}.[7, 11, 13] Notably, the 10 μM SACLAC-first treatment group (cf. Scheme 1B in the main text) shows the most distinct PC1 loading profile (upper trace in Figure S3H). Here, the pronounced ring deformation mode of phenylalanine shifted from 998 cm^{-1}[4, 11, 14, 17] to 1010 cm^{-1}.[4] The tryptophan vibration at 415 cm^{-1}[7, 11, 13] was not observed, and new bands at 932 and 534 cm^{-1} occurred, which can be attributed to Proline/Valine C-C stretching[4, 13] and cholesterol[4, 17] bands, respectively. These unique changes indicate that a higher level of the ceramide accumulation during organelle maturation that is the case with this incubation scheme (Scheme 1B in the main text) leads to remodeling of the molecular organization in the endolysosomes, particularly regarding the exposure of amino acid side chains and lipid membrane stability. In contrast, complex endolysosomes that formed before SACLAC treatment (following Scheme 1A in the main text) exhibited a relatively stable response to the higher ceramide levels.

The PC2 loadings exhibit greater variability across the different incubation conditions with the inhibitor and the optical nanoprobes. When 3T3 cells were treated with SACLAC after the gold nanoprobes (Scheme 1A in the main text), the AC activity at lower inhibitor dose (bottom trace in Figure S3B) leads to differences with respect to lipid acyl chain stretching modes at 1112 and 1094 cm^{-1},[4, 17, 19] and vibrations of tryptophan at 1339 cm^{-1}[7, 11, 13, 17] and disulfide S-S bonds at 585 cm^{-1}.[13] In contrast, at AC inhibition by 10 μM SACLAC (bottom trace in Figure S3D, following Scheme 1A in the main text) signals of

cholesterol at 425 cm^{-1},[4] of tryptophan at 1355 cm^{-1},[7, 11, 13, 17] and another S-S bond frequency of 558 cm^{-1} [9, 13] are representative of the molecular variation that is induced by the ceramide increase. The loadings of PC2 of the inhibitor-first treatments (following Scheme 1B in the main text) with both low and high SACLAC dose resemble one another (bottom traces in Figure S3F and S3H), e.g., with respect to differences in the lipid CH_2 deformation mode at 1440 cm^{-1},[4, 7, 11, 17] a C-C stretching signal at 1114 cm^{-1},[4, 17, 19] and a S-S stretching band at 459 cm^{-1}.[7, 13] This indicates that the effects of different inhibitor dose are more heterogenous in complex endolysosomes that have formed before AC enzyme inhibition.

Principal component analysis of spectra from cells exposed to additional ASM

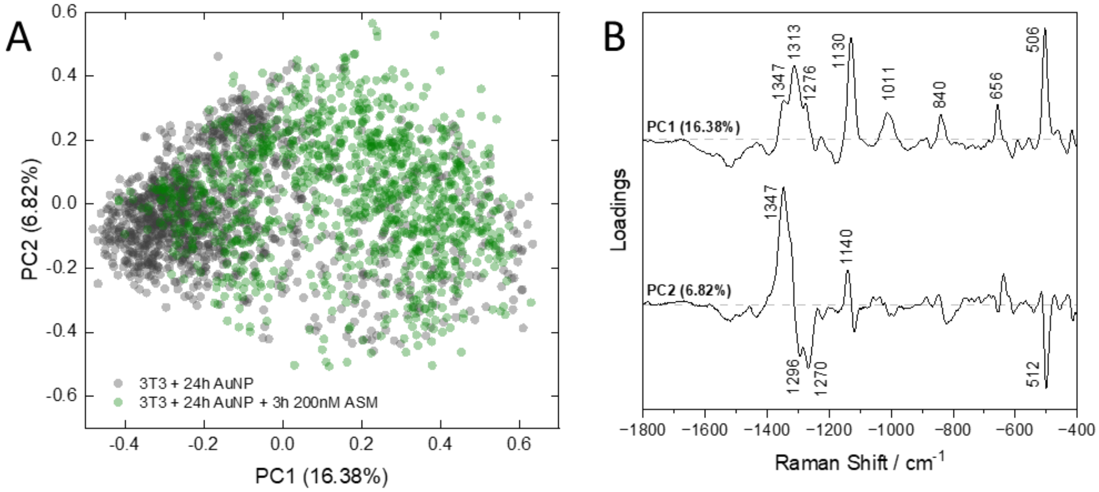

Figure S4. (A) Scores and **(B)** loadings of PC1 and PC2 of the principal component analysis (PCA) applied to a data set of 3T3 cells incubated with 200 nM ASM for 3 h after incubation with gold nanoparticles for 24 h and the control group without ASM addition. The analysis used the full spectral from 400 -1800 cm^{-1}.

Initial analysis of the data from ASM treated cells and their controls indicates substantial overlap and no clear separation of the data according to the two different sample sets in the score plot of the first two components (Figure S4A). In agreement with this shared variation, the first component is dominated by spectral features that vary both within the ASM-treated cells and also the control samples, respectively (Figure S4B, upper trace). The spectral differences closely resemble those identified in the PCA loadings of SACLAC treatments (upper traces in Figure S3B, S3D, S3F and S3H), with the exception of a tryptophan mode at 415 cm^{-1} [7, 11, 13] that is replaced by one at 1347 cm^{-1}.[7, 11, 13, 17] Such similarity indicates a common physiological response in endolysosomes to the ceramide enrichment for acid ceramidase inhibition and ASM addition. The subtle differences observed under ASM treatment were less diverse than those under SACLAC treatments, which might be attributed to the shorter exposure duration to ASM.

Random Forest based analysis of SERS spectra from 3T3 cells treated with SACLAC and ASM

Table S2. Performance of RF analysis to discriminate the spectra of 3T3 cells with different SACLAC and ASM treatments from spectra of their respective control group only incubated with gold nanoparticles (AuNPs)

Cells	Conditions	Accuracy	
		Training	Test
3T3	24h AuNPs + 24h 1 µM SACLAC	89.14%	91.41%
	24h AuNPs + 24h 10 µM SACLAC	84.53%	85.10%
	24h 1 µM SACLAC + 24h AuNPs	91.21%	90.28%
	24h 10 µM SACLAC + 24h AuNPs	89.23%	86.09%
	24h AuNPs + 3h 200 nM ASM	91.15%	91.74%

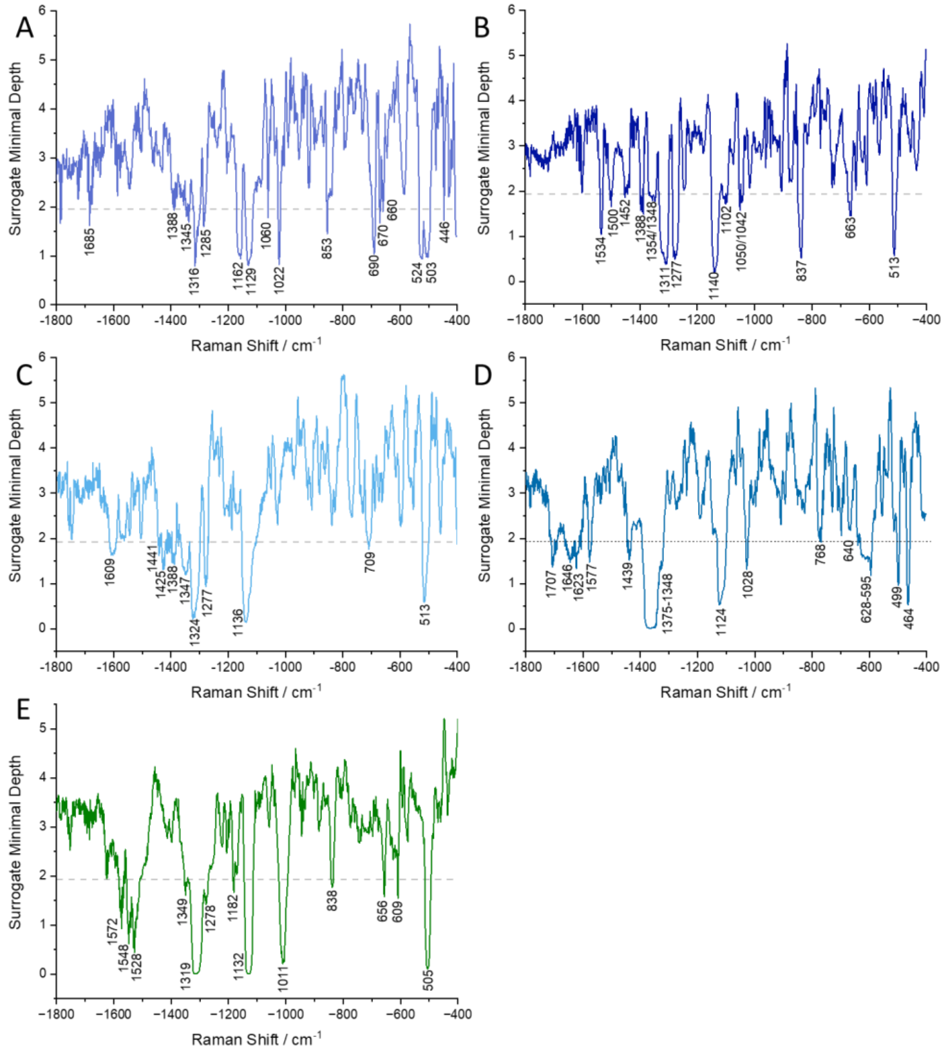

Figure S5. Selection of important bands of SERS spectra from 3T3 cells by the importance parameter surrogate minimal depth (SMD) for inhibition of AC by SACLAC (A-D) and addition of ASM (E).

SMD for data sets of cells treated with **(A)** 1 µM SACLAC and **(B)** 10 µM SACLAC for 24 h after incubation with gold nanoparticles for 24 h (Scheme 1A, cf. main text), with **(C)** 1 µM SACLAC and **(D)** 10 µM SACLAC for 24 h, prior to 24 h incubation of gold nanoparticles (Scheme 1B, cf. main text), and with **(E)** 200 nM ASM for 3 h after incubation with gold nanoparticles for 24 h (Scheme 1C, cf. manuscript). Spectral variables are selected if they have importance values below the threshold shown as a dashed line in each plot.

The analysis of surrogate minimal depth (SMD) in Figure S5 shows features that are specific of the different enzyme manipulations, which is different from the PCA results (Figure S4), where distinctions could not be identified. Comparison of the results using different SACLAC concentrations shows that when a lower concentration of SACLAC is used after incubation with the gold nanoprobes (Figure S5A, Scheme 1A in the main text) the amide I band at 1685 cm^{-1} [8, 13] and multiple disulfide vibrations in 500-690 cm^{-1} [2, 7, 9, 11, 13, 17] are important bands in the separation of treated from control samples. The higher concentration of SACLAC (Figure S5B) results in more differences in the amide II components at 1534 cm^{-1} [4, 11, 13, 18] and 1500 cm^{-1},[13] and with respect to the bands at 1452 cm^{-1} assigned to CH_2 deformation, [4, 8, 13, 17]as well as signals at 1102 cm^{-1} and 1050 cm^{-1} of C-C stretching modes.[4, 9, 17, 18] The latter indicate differences in lipid packing and intrachain conformation.

When 1 µM SACLAC was first given to the cells and probed endoloysosomes form afterwards (Figure S5C, Scheme 1B in the main text), C=C stretching vibrations of aromatic amino acids at 1609 cm^{-1} [4, 14, 20] and a lipid CH_3 bending mode at 1388 cm^{-1} [4] were selected as important, very different from the loadings of the corresponding PCA that indicated important changes at 1012 cm^{-1}, 834 cm^{-1} and 415 cm^{-1} (Figure S3F). The selection of the bands at 709 cm^{-1} [20] and 513 cm^{-1} of disulfide bands [2, 7, 11, 13, 17] and at 1347 cm^{-1} of the tryptophan suggests that endolysosomal proteins may undergo more subtle structural modifications during progressing ceramide accumulation. The features that were selected as important classifiers in the analysis of spectra measured after the exposure of the cells to the higher, 10 µM SACLAC concentration prior to the addition of AuNPs (Scheme 1B in the main text) can be assigned to several amide components, e.g. at 1707 cm^{-1}, 1646 cm^{-1}, and 1577 cm^{-1} [11, 13] and to signals of aromatic amino acids, e.g., at 1623 cm^{-1},[4] 768 cm^{-1},[4, 10, 13] and 621 cm^{-1} [4, 8, 12], which were not apparent in the respective PCA loadings (Figure S3H). The importance of phosphatidylinositol and cholesterol signals at 595 cm^{-1} [4] and 613 cm^{-1}, [4] respectively, identified in the broad SMD feature in 595-629 cm^{-1}, indicate that reorganization processes in the membrane must occur during the stronger enzyme inhibition. Also under these conditions, the spectral features that are important in the separation of the treated and control cells differ from the variation indicated in the PCA loadings. Compared to the experiments where enzyme inhibition starts after

formation of the endolysosomes (Figure S5A and S5B), the absent contributions of lipid C-C stretching in 1050-1120 cm^{-1} [4, 9, 17-19] when the endolysosomes develop during enzyme inhibition at both SACLAC concentrations (Figure S5C and S5D) suggest fewer differences in lipid membrane disorder during gradual ceramide accumulation. Notably, the cholesterol-related signals only appeared as important features at 446 cm^{-1} [17] (Figure S5B) and at 613 cm^{-1} [4] (Figure S5D), respectively, when the inhibitor SACLAC was applied at the higher concentration of 10 μM. This suggests that the ceramide-induced changes in membrane stability require a certain concentration of the lipid.

Different from treatment of the cells with the AC inhibitor SACLAC, the selected spectral features in an RF analysis of the data from the cells supplemented with additional ASM (Figure S5E) are more similar to the variation indicated by the respective PCA loadings (Figure S4B). In addition, RF-SMD indicated important contributions by the amide II components at 1572 cm^{-1}, 1548 cm^{-1}, and 1528 cm^{-1} [4, 11, 13] to the discrimination of spectra from ASM enriched and control cells (Figure S5E). In agreement with the SMD results in the samples manipulated with the inhibitor (Figure S5A-S5D), the importance of amide vibrations revealed by SMD of the data from ASM-treated cells also suggests subtle changes in protein conformation that are induced by an increased ceramide level in endolysosomes to varied extent. Additional bands at 609 cm^{-1} and 1182 cm^{-1} assigned to cholesterol[4] and lipid phosphate[11, 17, 18] and related to lipid metabolism that is mediated by ASM, were also selected by the algorithm.

SERS data of cells incubated with ceramide at different concentrations

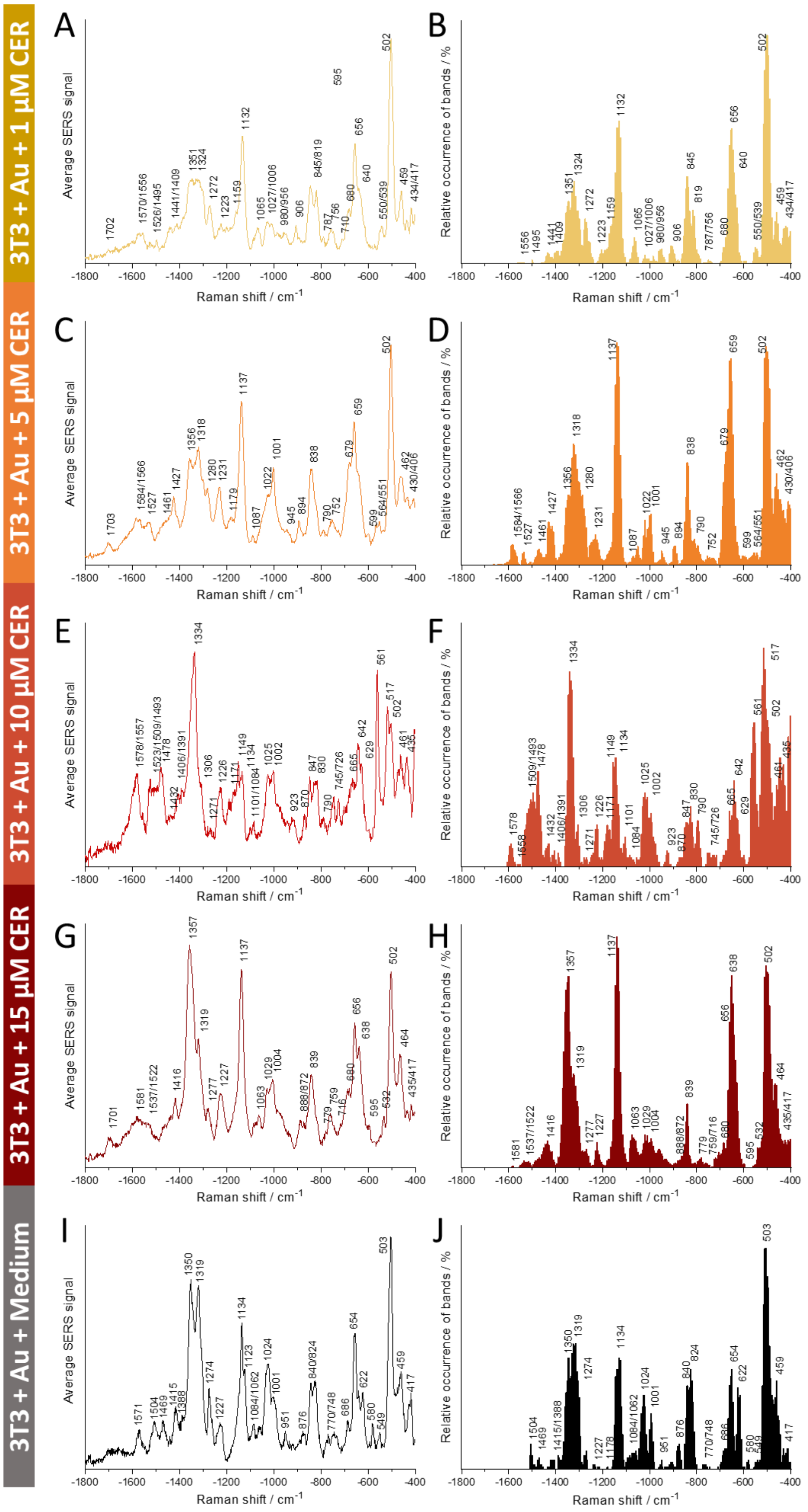

Figure S6. Average SERS spectra (A, C, E, G, I) and relative band occurrence (B, D, F, H, J) of the respective SERS data sets of 3T3 cells incubated with ceramide at a concentration of **(A, B)** 1 µM, **(C, D)** 5 µM, **(E, F)** 10 µM, and **(G, H)** 15 µM after incubation with gold nanoparticles for 24 h (Scheme 1D, cf.

manuscript), and **(I, J)** control samples that were incubated only with gold nanoparticles for 24 h. The data sets contain 237 (A, B), 502 (C, D), 294 (E, F), 517 (G, H) and 180 (I, J) SERS spectra, respectively. Excitation wavelength: 785 nm. Excitation intensity: 2.7×10^5 W cm^{-2}. Acquisition time: 1 s.

The average SERS spectra the cells treated with ceramide (cf. Scheme 1D in the main text) at concentrations of 1 µM, 5 µM, 10 µM, and 15 µM (Figure S6A, S6C, S6E and S6G) similarly shared primary vibrational bands associated with protein interactions with the spectra of the control samples (Figure S6I).

Despite several similarities, most of the spectra from cells that were exposed to ceramide shared several features across the four ceramide concentrations (Figure S6A, S6C, S6E and S6G) that were absent in the control sample (Figure S6I and S6J). The presence of bands assigned to the amide group at 1702 cm^{-1} [8] and 1525 cm^{-1},[13] to tyrosine at 640 cm^{-1},[8, 12, 14] and cholesterol at 434 cm^{-1} [17] imply changes in protein secondary structure, an exposure of typically buried protein domains, and an altered membrane lipid composition, independent of the concentration of applied ceramide. Treated cells also lack specific signals that are present in the spectra of the control samples. The absence of the amide II signal at 1504 cm^{-1} [13] and of the phenylalanine vibration at 622 cm^{-1} [4, 8, 12] indicates modified protein secondary structure, and absent lipid signals at 1469 cm^{-1},[4, 8, 13, 17] 1123 cm^{-1},[4, 17, 19] and 824 cm^{-1} [4] reveal a destabilization of membranes. The lack of the disulfide signals at 549 cm^{-1} and 580 cm^{-1} [9, 13] suggests fewer disulfide bridges, suggesting that more fragmented proteins interact with the nanoprobes.[13] The spectra obtained after adding ceramide at any of the concentrations generally exhibit an increased occurrence of signals assigned to C-C/C-N vibrations at 1135 cm^{-1} [7, 11] and to C-S bonds at 656 cm^{-1},[2, 7, 11, 17] as well as less frequently occurring signals of phenylalanine at 1025 cm^{-1} [4, 5, 9, 14] and 1003 cm^{-1} [4, 11, 14, 17] (Figure S6B, S6D, S6F and S6H).

Several spectral changes were also observed that varied for the different ceramide concentrations. The amide III component at 1225 cm^{-1}, characteristic of unordered protein structure[4] became more frequent than the amide III mode at 1277 cm^{-1} that is related to α-helical structures[4] at higher ceramide concentrations (10 µM and 15 µM, compare Figure S6F and S6H with S6B and S6D). This indicates changes in the secondary structures of endolysosomal proteins that depends on the concentration of the exogenous ceramide that the cells were exposed to. Furthermore, a disruption in the microenvironment of several amino acid residues was observed. As examples, the treatment of the cells with 10 µM ceramide led to a more frequent occurrence of a tryptophan band at the higher wavenumber of 1334 cm^{-1} (Figure S6F), compared to cells treated with 15 µM ceramide, that exhibited a much more frequent presence of

the tryptophan band at 1357 cm^{-1} [7, 11, 13, 17] (Figure S6H). The presence of C-S bands in 620-690 cm^{-1} [2, 4, 7, 8, 11, 12] increased differently in treated cells (Figure S6B, S6D, S6F and S6H) compared to the control samples, which could be attributed to protein unfolding to a different extent.[22]

The spectra of cells treated with 1 µM and 15 µM ceramide (Figure S6A and S6C) lack the symmetric C-C vibration at 1084 cm^{-1},[15, 19] whereas the asymmetric mode at 1065 cm^{-1} [15, 19] is rare in the spectra of cells treated with 5 µM and 10 µM ceramide (Figure S6C and S6E). This is different from the co-existence of both lipid bands in the spectra of the control samples (Figure S6I). In agreement with this, the CH_2 deformation mode in the range of 1420-1480 cm^{-1} [4, 7, 8, 11, 13, 16, 17] exhibits varied spectral shifts and occurrence patterns (Figure S6A to S6H), reflecting differences in membrane packing that vary for different concentrations of the applied ceramide. Moreover, significant changes in lipid composition were observed as evidenced by additional cholesterol band at 539 cm^{-1} [4, 17] upon 1 µM ceramide addition (Figure S6A), as well as new phosphoinositol markers[4, 17] at higher ceramide concentrations (Figure S6C, S6E and S6G, Table S1). Especially the treatment with 10 µM in Figure S6F shows a phosphoinositol band at 517 cm^{-1} [4] with a remarkably high relative occurrence.

Additionally, DNA/RNA bands at 790 cm^{-1} [4, 8] were found in the spectra of cells treated with 1 µM, 5 µM, and 10 µM ceramide (Figure S6A, S6C and S6E). They suggest that nucleic acids that undergo degradation or processing in the endolysosomes must be in close proximity to the nanoprobes due to an altered endolysosomal environment.

Overall, despite several similar features related to protein changes on membranes, the cells exposed to ceramide displayed interactions of different molecular constituents with the gold nanoprobes that depend on ceramide concentration. At the lowest concentration of 1 µM, mainly protein conformational alterations accounted for the predominant spectral differences between treated and untreated cells. The initial lipid changes, were observed with respect to the cholesterol signal, together with weakened phospholipid acyl chain vibrations. With an increased ceramide concentrations changes in the spectra indicate an altered lipid organization, and phosphoinositol signals revealed that lipid remodelling must have occurred. The presence of different phosphoinositol vibrations under exposure to higher ceramide levels of 10 µM and 15 µM suggest a rearrangement of membranes that is specific of these concentrations. These observed vibrational changes show an influence of the concentration of the ceramide that is taken up by the cells. This is consistent with previous findings in coronary artery endothelial cells[23] and adenocarcinoma HCT116 cells[24] that displayed reduced viability upon exposure to exogenous C16 ceramide in a dose-dependent manner.

Principal component analysis of SERS spectra from cells treated with ceramide

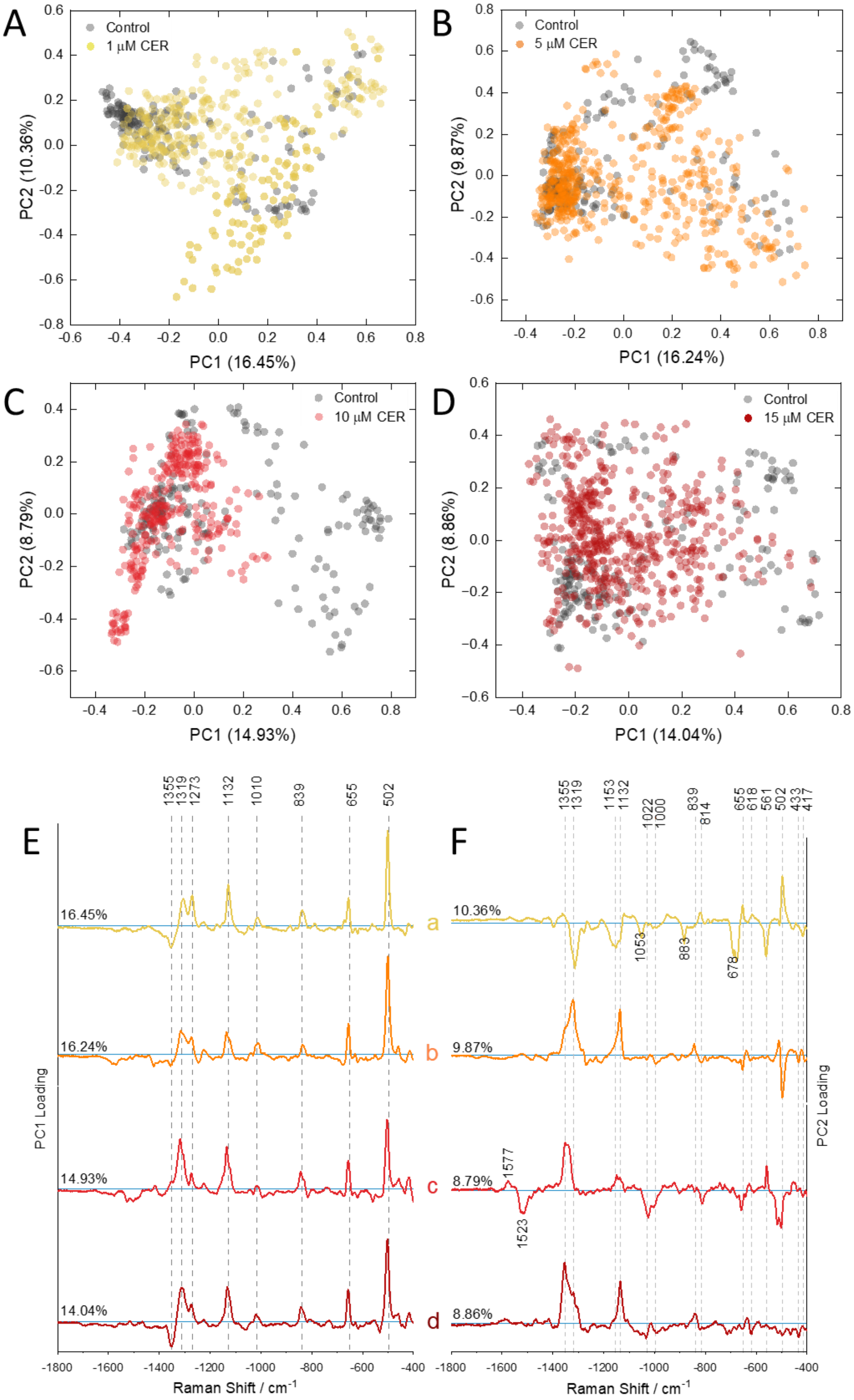

Figure S7. Principal component scores of the PCA applied to data sets of 3T3 cells incubated with gold nanoparticles for 24 h prior to incubation with ceramide at a concentration of **(A)** 1 μM, **(B)** 5 μM, **(C)** 10 μM, **(D)** 15 μM compared to the control group. **(E, F)** Loadings of PC1 and PC2 for all analyses of (a) 1 μM, (b) 5 μM, (c) 10 μM, (d) 15 μM). The analyses used the full spectral from 400 -1800 cm^{-1}.

The PCA score plots of exogenous ceramide treatments were generated for each pairwise comparison between control and treated cells(Figure S7A, S7B, S7C and S7D). Substantial overlap was observed between the control and treated groups across all ceramide concentrations, indicating that the spectral variance contained in PC1 and PC2 does not clearly separate both groups.

The loadings of the first two principal components, PC1 and PC2(Figure S7E and S7F) show key spectral features that contribute to the observed variance. The loadings of PC1 exhibited high similarity across all four comparisons. In agreement with the spectra and statistical analysis of band occurrence (Figure S6), the bands assigned to protein backbone C-C/C-N bond at 1132 cm^{-1},[7, 11] disulfide C-S bond at 655 cm^{-1} [2, 7, 11, 17] and S-S bond at 502 cm^{-1},[2, 7, 11, 13, 17] tyrosine at 839 cm^{-1} [4, 6, 8, 17] and phenylalanine at 1010 cm^{-1} [4, 11, 14, 17] appear across all experimental conditions with comparable contributions, suggesting that conformational changes of proteins in the endolysosomes distinguish ceramide-treated cells from the control group. Slight differences were observed from amide III vibration at 1273 cm^{-1},[4, 11, 13] tryptophan at 1355 cm^{-1},[7, 11, 13, 17] and lipid CH_2/CH_3 deformation at 1319 cm^{-1},[11, 18] consistent with the findings of concentration-dependent changes of protein conformation and lipid packing when ceramide was increased by application of SACLAC.

The loadings of PC2 (Figure S7F) shows more variations that were characteristic of the different ceramide concentration. Overall, PC2 captured several ceramide-induced differences that can be associated with amino acid side chains. They include the contributions of the phenylalanine ring breathing vibration at 1000 cm^{-1} [4, 11, 14, 17] and the C-H in-plane bending vibration at 1022 cm^{-1}. [4, 5, 9, 14]. The tryptophan band at 561 cm^{-1} [4] is absent in the comparisons of cells treated with 5 and 15 μM ceramide with the control group (trace b and c in Figure S7F). For cells treated with 1 μM ceramide, another C-S band at 678 cm^{-1} [11, 13] was identified (trace a in Figure S7F), suggesting distinct interactions of cysteine residues. Additional differences were exhibited in a signal at 883 cm^{-1} that we assign to a tryptophan ring deformation mode[4] and at 1053 cm^{-1} of a lipid C-C stretch.[4, 9, 17, 18] Both suggest that the exposure of the cells to ceramide at the lowest concentration of 1 μM may alter the microenvironment of amino acids and nearby membranes in a different way compared to an exposure to ceramide at higher concentration.

Random Forest based analysis of SERS spectra from 3T3 cells treated with ceramide

Table S3. Performance of RF analysis to discriminate the spectra of 3T3 cells treated with ceramide at different concentrations

Prediction	True				
	control	1 µM	5 µM	10 µM	15 µM
control	107	0	2	0	0
1 µM	3	382	10	2	17
5 µM	16	14	424	5	17
10 µM	31	17	16	260	2
15 µM	23	48	50	27	481
Sensitivity	59.44%	82.86%	84.46%	88.44%	93.04%

Only 59.44% of the spectra from the control samples were classified correctly, in agreement with the high spectral similarity between untreated and ceramide-treated samples (Figure S6) as well as the global overlap in the PCA score plots (Figure S7A, S7B, S7C and S7D).

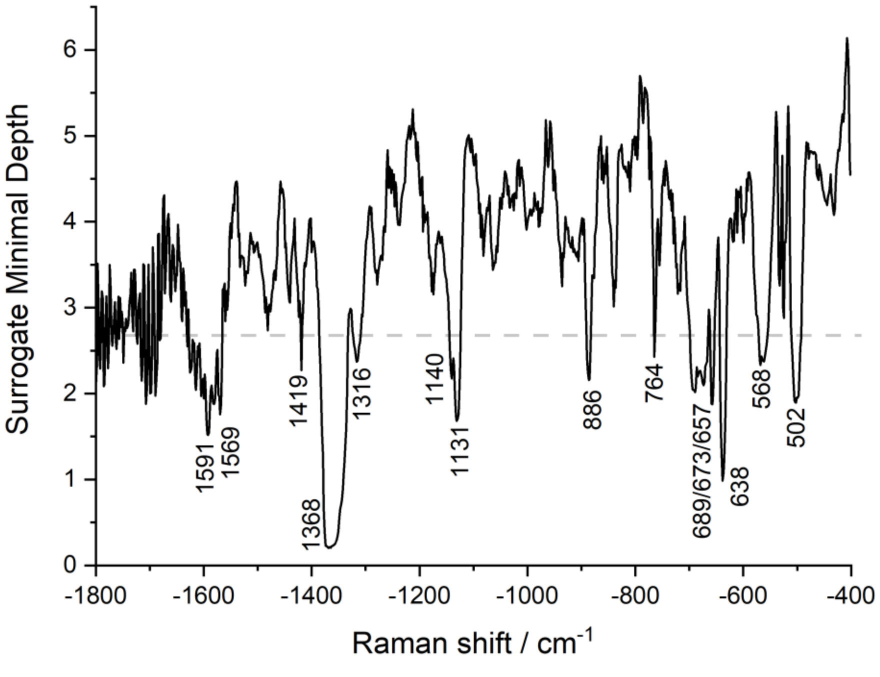

Figure S8. Selection of important bands by the importance parameter surrogate minimal depth (SMD) of SERS spectra from 3T3 cells incubated with ceramide at different concentrations after incubation with gold nanoparticles for 24 h. Spectral variables are selected if they have importance values below the threshold shown as a dashed line.

The results of SMD of spectral variables reveal the features that are important in order to distinguish cells treated with different concentrations of ceramide in a random forest analysis (Figure S8). Protein backbone (1140 and 1131 cm^{-1}),[7, 11] disulfide group (657 and 502 cm^{-1}),[2, 7, 11, 13, 17] tryptophan mode (1363 cm^{-1})[7, 11, 13, 17] and lipid CH_2/CH_3 deformation vibrations (1316 cm^{-1})[11, 18] were s identified as major contributors, based on their SMD (Figure S8). These signals were also apparent in the PC1 loadings of the respective PCAs (Figure S7E and S7F), suggesting that the main differences in the dataset are also relevant for distinguishing between the effects of different ceramide concentrations. Unlike the results of PCA, however, SMD selects phenylalanine and amide II vibrations at 1591 cm^{-1} [4, 14, 20] and 1569 cm^{-1} [11, 13] (Figure S8), respectively, rather than amide III bands at 1273 cm^{-1} [4, 11, 13] (Figure S7E). In addition, multiple C-S stretching modes (690 cm^{-1}, 673 cm^{-1}, and 638 cm^{-1})[2, 4, 7, 8, 11, 12] and tryptophan bands (764 cm^{-1} and 568 cm^{-1})[4, 10, 13] are selected (Figure S8), underpinning the key role of sulfur-containing and aromatic amino acids for the separation of spectra obtained from experiments with different ceramide concentrations. Furthermore, a CH_2 twisting vibration signal at 1419 cm^{-1} [11, 13, 16] was observed as important spectral variable, which indicates an effect on lipid reorganization. Interestingly, although the bands related to cholesterol and phospholipids appeared visually distinct in the spectral data (Figure S6), they do not contribute significantly to overall group variation.

Soft X-ray tomograms of 3T3 cells treated with SACLAC and ASM

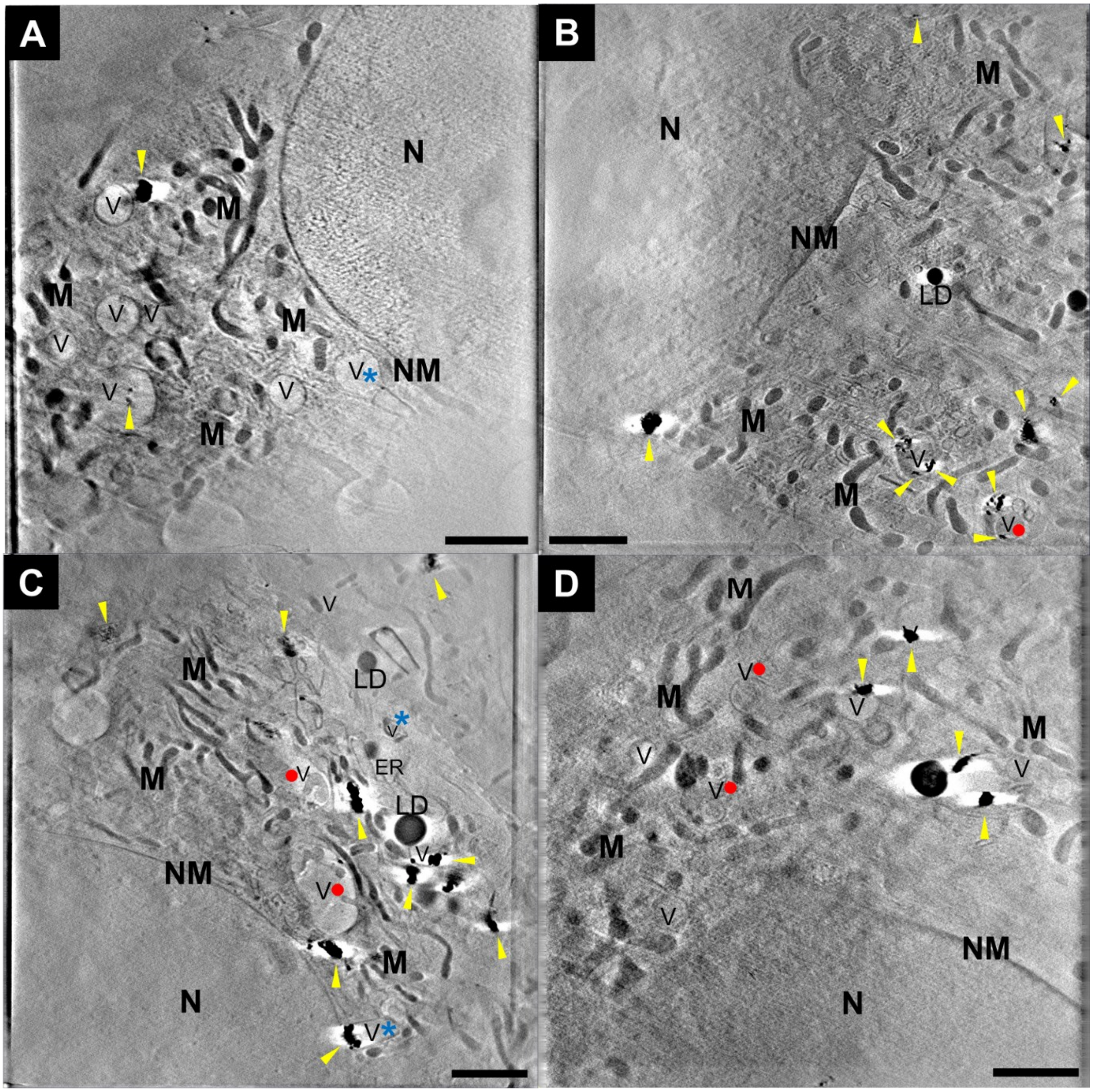

Figure S9. Reconstructed X-ray tomograms of 3T3 cells incubated with **(A)** 1 µM SACLAC and **(B)** 10 µM SACLAC for 24 h after incubation with gold nanoparticles for 24 h (Scheme 1A, cf. main text), **(C)** 3T3 cells incubated with gold nanoparticles for 24 h after seeding in the culture medium with 10 µM SACLAC for 24 h (Scheme 1B, cf. main text), **(D)** 3T3 cells incubated with 200 nM ASM for 3 h after incubation with gold nanoparticles for 24 h (Scheme 1C, cf. main text). M: mitochondrion, V: vesicle, NM: nuclear membrane, N: nucleus, LD: lipid droplets, ER: endoplasmic reticulum. Gold nanostructures are marked with yellow arrowheads. Red dots and blue asterisks mark the affected vesicles. Scale bar: 2 µm.

Reference

(1) Garidel, P.; Folting, B.; Schaller, I.; Kerth, A. The microstructure of the stratum corneum lipid barrier: Mid-infrared spectroscopic studies of hydrated ceramide:Palmitic acid:Cholesterol model systems. *Biophys. Chem.* **2010**, *150* (1-3), 144-156. DOI: 10.1016/j.bpc.2010.03.008.
(2) Lopez-Tobar, E.; Hernández, B. n.; Ghomi, M.; Sanchez-Cortes, S. Stability of the disulfide bond in cystine adsorbed on silver and gold nanoparticles as evidenced by SERS data. *J. Phys. Chem. C* **2013**, *117* (3), 1531-1537. DOI: 10.1021/jp3112606.
(3) Madzharova, F.; Heiner, Z.; Gühlke, M.; Kneipp, J. Surface-enhanced Hyper-Raman spectra of adenine, guanine, cytosine, thymine, and uracil. *The Journal of Physical Chemistry. C, Nanomaterials and Interfaces* **2016/07/07**, *120* (28), 15415-15423. DOI: 10.1021/acs.jpcc.6b02753.
(4) Movasaghi, Z.; Rehman, S.; Rehman, I. U. Raman spectroscopy of biological tissues. *Appl. Spectrosc. Rev.* **2007**, *42* (5), 493-541. DOI: 10.1080/05704920701551530.
(5) Notingher, I.; Green, C.; Dyer, C.; Perkins, E.; Hopkins, N.; Lindsay, C.; Hench, L. L. Discrimination between ricin and sulphur mustard toxicity in vitro using Raman spectroscopy. *J. R. Soc. Interface* **2004**, *1* (1), 79-90. DOI: 10.1098/rsif.2004.0008.
(6) Parker, F. S. *Applications of Infrared, Raman, and resonance Raman spectroscopy in biochemistry*; Springer Science & Business Media, 1983.
(7) Szekeres, G. P.; Werner, S.; Guttmann, P.; Spedalieri, C.; Drescher, D.; Živanović, V.; Montes-Bayón, M.; Bettmer, J.; Kneipp, J. Relating the composition and interface interactions in the hard corona of gold nanoparticles to the induced response mechanisms in living cells. *Nanoscale* **2020**, *12* (33), 17450-17461. DOI: 10.1039/d0nr03581e.
(8) Pezzotti, G. Raman spectroscopy in cell biology and microbiology. *J. Raman Spectrosc.* **2021**, *52* (12). DOI: 10.1002/jrs.6204.
(9) Rothschild, K. J.; Andrew, J. R.; Grip, W. J. D.; Stanley, H. E. Opsin structure probed by Raman spectroscopy of photoreceptor membranes. *Science* **1976**, *191* (4232), 1176-1178. DOI: 10.1126/science.1257742.
(10) Simon, I.; Hedesiu, M.; Virag, P.; Salmon, B.; Tarmure, V.; Baciut, M.; Bran, S.; Jacobs, R.; Falamas, A. Raman micro-spectroscopy of dental pulp stem cells: An approach to monitor the effects of cone beam computed tomography low-dose ionizing radiation. *Anal. Lett.* **2019**, *52* (7), 1097-1111. DOI: 10.1080/00032719.2018.1516771.
(11) Spedalieri, C.; Szekeres, G. P.; Werner, S.; Guttmann, P.; Kneipp, J. Intracellular optical probing with gold nanostars. *Nanoscale* **2021**, *13* (2), 968-979. DOI: 10.1039/d0nr07031a.
(12) Stone, N.; Kendall, C.; Smith, J.; Crow, P.; Barr, H. Raman spectroscopy for identification of epithelial cancers. *Faraday Discuss.* **2004**, *126*, 141-157. DOI: 10.1039/b304992b.
(13) Szekeres, G. P.; Montes-Bayón, M.; Bettmer, J.; Kneipp, J. Fragmentation of proteins in the corona of gold nanoparticles as observed in live cell surface-enhanced Raman scattering. *Anal. Chem.* **2020**, *92* (12), 8553-8560. DOI: 10.1021/acs.analchem.0c01404.
(14) Tang, S.; Gao, S.; Xu, J.; Zheng, M.; Huang, Y.; Yu, Y.; Lin, J. A novel serum protein purification technique combined with surface-enhanced Raman spectroscopy for liver cancer detection. *Spectrosc. Lett.* **2021**, *54* (2), 113-121. DOI: 10.1080/00387010.2020.1867186.
(15) Tfayli, A.; Guillard, E.; Manfait, M.; Baillet-Guffroy, A. Molecular interactions of penetration enhancers within ceramides organization: A Raman spectroscopy approach. *Analyst* **2012**, *137* (21), 5002. DOI: 10.1039/c2an35220f.
(16) Živanović, V.; Milewska, A.; Leosson, K.; Kneipp, J. Molecular structure and interactions of lipids in the outer membrane of living cells based on surface-enhanced Raman scattering and liposome models.

Anal. Chem. **2021**, *93* (29), 10106-10113. DOI: 10.1021/acs.analchem.1c00964.
(17) Živanović, V.; Seifert, S.; Drescher, D.; Schrade, P.; Werner, S.; Guttmann, P.; Szekeres, G. P.; Bachmann, S.; Schneider, G.; Arenz, C.; Kneipp, J. Optical nanosensing of lipid accumulation due to enzyme inhibition in live cells. *ACS Nano* **2019**, *13* (8), 9363-9375. DOI: 10.1021/acsnano.9b04001.
(18) Feng, Y.; Gärber, F.; Saied, E. M.; Spedalieri, C.; Kochovski, Z.; Werner, S.; Pratsch, C.; Arenz, C.; Seifert, S.; Kneipp, J. SERS spectra indicate the molecular effects of 7-nitrobenz-2-oxa-1,3-diazole (nbd) on living cells. *J. Phys. Chem. C* **2024**, *128* (46), 19722-19735. DOI: 10.1021/acs.jpcc.4c05260.
(19) Guillard, E.; Tfayli, A.; Manfait, M.; Baillet-Guffroy, A. Thermal dependence of Raman descriptors of ceramides. Part ii: Effect of chains lengths and head group structures. *Anal. Bioanal. Chem.* **2011**, *399* (3), 1201-1213. DOI: 10.1007/s00216-010-4389-x.
(20) Podstawka, E.; Ozaki, Y.; Proniewicz, L. M. Part III: Surface-enhanced Raman scattering of amino acids and their homodipeptide monolayers deposited onto colloidal gold surface. *Appl. Spectrosc.* **2005**, *59* (12), 1516-1526. DOI: 10.1366/000370205775142520.
(21) Dos Santos, D. P.; Temperini, M. L. A.; Brolo, A. G. Intensity fluctuations in single-molecule surface-enhanced Raman scattering. *Acc. Chem. Res.* **2019**, *52* (2), 456-464. DOI: 10.1021/acs.accounts.8b00563.
(22) Bazylewski, P.; Divigalpitiya, R.; Fanchini, G. In situ Raman spectroscopy distinguishes between reversible and irreversible thiol modifications in l-cysteine. *RSC Advances* **2017**, *7* (5), 2964-2970. DOI: 10.1039/c6ra25879d.
(23) Zietzer, A.; Jahnel, A. L.; Bulic, M.; Gutbrod, K.; Düsing, P.; Hosen, M. R.; Dörmann, P.; Werner, N.; Nickenig, G.; Jansen, F. Activation of neutral sphingomyelinase 2 through hyperglycemia contributes to endothelial apoptosis via vesicle-bound intercellular transfer of ceramides. *Cell. Mol. Life Sci.* **2022**, *79* (1), 1-20. DOI: 10.1007/s00018-021-04049-5.
(24) Rénert, A.-F.; Leprince, P.; Dieu, M.; Renaut, J.; Raes, M.; Bours, V.; Chapelle, J.-P.; Piette, J.; Merville, M.-P.; Fillet, M. The proapoptotic c16-ceramide-dependent pathway requires the death-promoting factor btf in colon adenocarcinoma cells. *J. Proteome Res.* **2009**, *8* (10), 4810-4822. DOI: 10.1021/pr9005316.
